# DNA–Methylome Analysis of Mouse Intestinal Adenoma Identifies a Tumour-Specific Signature That Is Partly Conserved in Human Colon Cancer

**DOI:** 10.1371/journal.pgen.1003250

**Published:** 2013-02-07

**Authors:** Christina Grimm, Lukas Chavez, Mireia Vilardell, Alexandra L. Farrall, Sascha Tierling, Julia W. Böhm, Phillip Grote, Matthias Lienhard, Jörn Dietrich, Bernd Timmermann, Jörn Walter, Michal R. Schweiger, Hans Lehrach, Ralf Herwig, Bernhard G. Herrmann, Markus Morkel

**Affiliations:** 1 Max Planck Institute for Molecular Genetics, Department of Vertebrate Genomics, Berlin, Germany; 2 Charité Universitätsmedizin Berlin, Department of Rheumatology, Berlin, Germany; 3 Max Planck Institute for Molecular Genetics, Department of Developmental Genetics, Berlin, Germany; 4 Universität des Saarlandes, FR 8.3 Biowissenschaften, Genetik/Epigenetik Campus, Saarbrücken, Germany; 5 Max Planck Institute for Molecular Genetics, Next Generation Sequencing Core Facility, Berlin, Germany; 6 Charité Universitätsmedizin Berlin, Institute for Medical Genetics, Berlin, Germany; 7 Charité Universitätsmedizin Berlin, Laboratory of Molecular Tumor Pathology, Berlin, Germany; Friedrich Miescher Institute for Biomedical Research, Switzerland

## Abstract

Aberrant CpG methylation is a universal epigenetic trait of cancer cell genomes. However, human cancer samples or cell lines preclude the investigation of epigenetic changes occurring early during tumour development. Here, we have used MeDIP-seq to analyse the DNA methylome of APC^Min^ adenoma as a model for intestinal cancer initiation, and we present a list of more than 13,000 recurring differentially methylated regions (DMRs) characterizing intestinal adenoma of the mouse. We show that Polycomb Repressive Complex (PRC) targets are strongly enriched among hypermethylated DMRs, and several PRC2 components and DNA methyltransferases were up-regulated in adenoma. We further demonstrate by bisulfite pyrosequencing of purified cell populations that the DMR signature arises *de novo* in adenoma cells rather than by expansion of a pre-existing pattern in intestinal stem cells or undifferentiated crypt cells. We found that epigenetic silencing of tumour suppressors, which occurs frequently in colon cancer, was rare in adenoma. Quite strikingly, we identified a core set of DMRs, which is conserved between mouse adenoma and human colon cancer, thus possibly revealing a global panel of epigenetically modified genes for intestinal tumours. Our data allow a distinction between early conserved epigenetic alterations occurring in intestinal adenoma and late stochastic events promoting colon cancer progression, and may facilitate the selection of more specific clinical epigenetic biomarkers.

## Author Summary

The formation and progression of tumours to metastatic disease is driven by two major mechanisms, i.e. genetic alterations that activate oncogenes or inactivate tumour suppressor genes, and changes in the epigenome that cause variations in the expression of the genetic information. A deeper understanding of the interaction between the genetic and epigenetic mechanisms is critical for the selection of tumour biomarkers and for the future development of therapies. Human tumour specimens and cell lines contain a plethora of genetic and epigenetic changes, which complicate data analysis. In contrast, mouse tumour models such as the APC^Min^ mouse used in this study arise by a single initiating genetic mutation, yet share key traits with human cancer. Here we show that mouse adenomas acquire a multitude of epigenetic alterations, which are recurring in mouse adenoma and in human colon cancer, representing early and advanced tumours, respectively. The use of a mouse model thus allowed us to uncover a sequence of epigenetic changes occurring in tumours, which may facilitate the identification of novel clinical colon cancer biomarkers.

## Introduction

Epigenetic mechanisms play critical roles in controlling the cellular transcript repertoire and ultimately cellular phenotypes. The methylation of cytosine bases of CpG dinucleotides, as well as the covalent modification of histone proteins represent major target sites of the protein complexes involved in epigenetic control. Epigenetic histone and DNA codes are established and interpreted in a combinatorial fashion, and both interact in the short-term regulation of gene activity and in the establishment of long-term epigenetic memory, such as heterochromatin formation and gene silencing [1], [2]. The Polycomb Repressive Complexes (PRC1 and PRC2) and Trithorax Group Complexes (TrxG) are major histone modifying protein complexes setting repressive or activating marks, respectively, while CpG methylation patterns are set and propagated by DNA methlytransferases (DNMTs). A major role of the PRC2 complex in development is to tag gene regulatory sequences with a specific tri-methyl mark on lysine 27 of histone 3 (H3K27me3), resulting in short-term transcriptional repression [3]. Moreover, PRC2 complexes can also interact with DNMTs, and initiate long-term silencing of genes via de-novo CpG methylation [4]. By default, non-transformed cells are characterized by high genome-wide CpG methylation, with the exception of CpG islands (CGIs), which are mostly unmethylated [1], [2], [5].

Tumour cells contain aberrant epigenomes [4], [6]–[12]. Tumours are characterized by general genomic hypomethylation of CpGs, while CGIs are hypermethylated [13]. It has been found that histone modification patterns of tumour cells resemble those found in embryonic stem cells and likely guide CpG methylation [14]–[18]. This epigenetic pattern of tumour cells has been associated with uncontrolled PRC2 activity [6], [15], [19]–[21]. As a consequence of the deregulation of epigenetic control mechanisms, many types of cancer display epigenetic silencing of tumour suppressor genes, which interact with genetic mutations in establishing the tumour phenotype.

Intestinal tumours of humans and mice usually initiate via hyperactivation of Wnt/beta-catenin signalling, which is often caused by genetic loss of the tumour suppressor APC [22], [23]. Additional genetic and epigenetic alterations are involved in the gradual loss of tissue homeostasis during tumour progression, among them epigenetic silencing of the tumour suppressor *Cdkn2a* (coding for a cell cycle and apoptosis control gene), *Dkk1* and *Sfrp* family genes (coding for Wnt antagonists) or *Hic1* (coding for a developmental control gene) [24], [25]. How and when colon cancer-specific methylation patterns form is largely unknown. This is in contrast to genetic mutations, of which many have been linked to specific stages of tumour progression [22].

Here we have utilized the APC^Min^ mouse model to characterize early steps of epigenetic modifications in intestinal cancer. APC^Min^ mice form multiple intestinal adenomas following the somatic loss of functional APC, similar to the initiation of a large majority of sporadic human colon cancers and to familial colon cancer syndromes [22], [23]. We report a comprehensive catalogue of differentially methylated regions (DMRs), which form *de novo* and consistently in APC^Min^ adenoma. Hypermethylated DMRs were found prevalently at sites of PRC2 activity, but silencing of tumour suppressors was rarely observed in adenoma. The comparison to human colon cancer samples identified a core epigenetic map of intestinal cancer DMRs, which is conserved between mice and humans.

## Results

### MeDIP-seq analysis of APC^Min^ adenomas reveals a large number of DMRs

**Figure 1:**
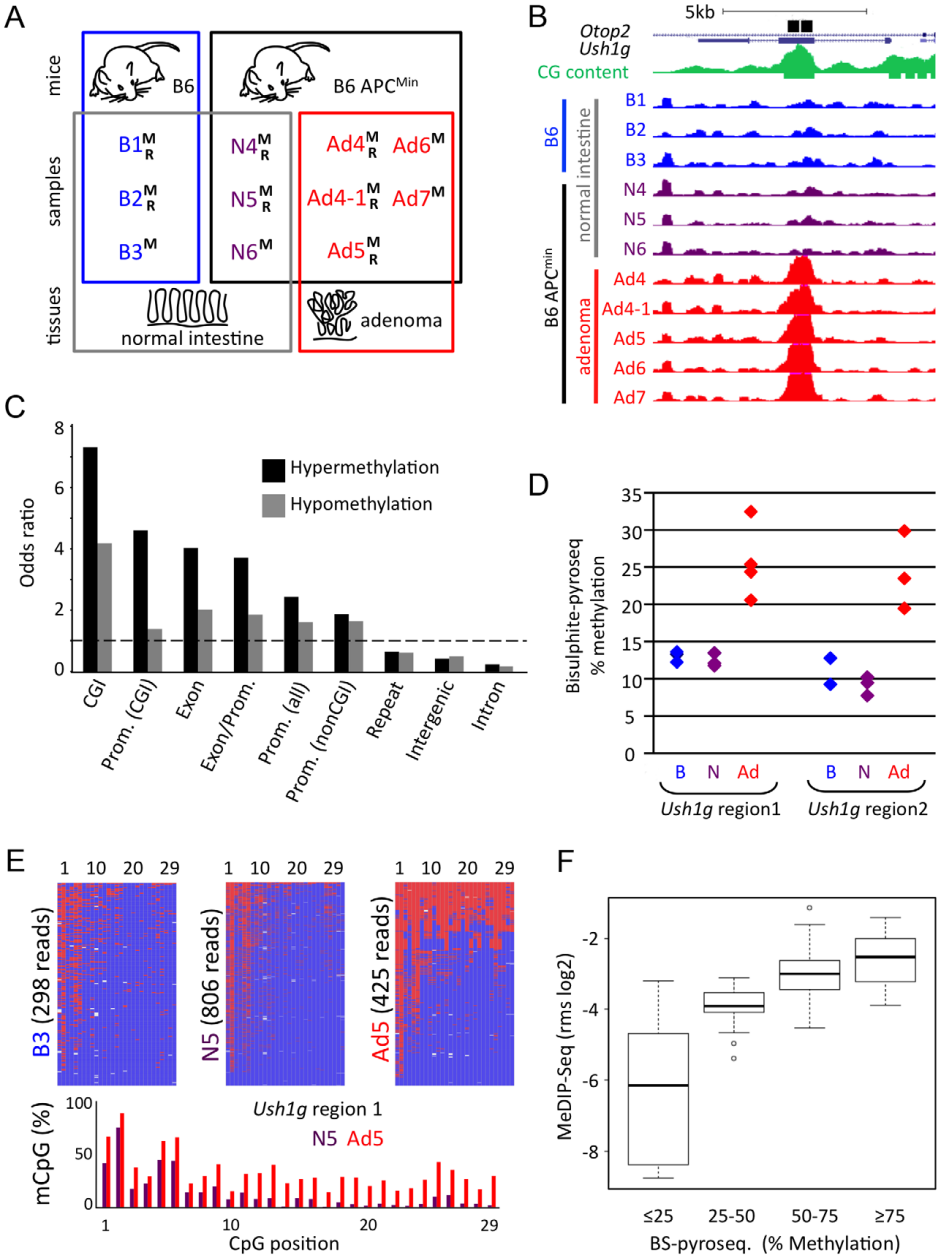
Generation and validation of genome-wide CpG methylation maps of APC^Min^ mouse normal and adenoma tissues. a) Summary of tissue samples used for genome-wide analyses. B6 wildtype (B) and isogenic B6-APC^Min^ (APC^Min^)mice were employed for MeDIP-seq (M) and RNA-seq (R) of normal intestinal tissue (B, N) and intestinal adenoma (Ad). b) Visualisation of the adenoma-hypermethylated DMR in *Ush1g*, using the UCSC browser. Maximal height for visualization was set to rpm = 2 for all MeDIP-seq tracks. Black bars, regions that were validated by SIRPH or bisulfite-pyrosequencing (see below, d, e); green, CpG density; blue, purple, red: MeDIP-seq tracks of B6 mouse normal intestine, APC^Min^ mouse normal intestine, and APC^Min^ adenoma, respectively. Mice/samples are numbered consecutively. c) Distribution of DMRs in different subgenomic compartments. Odds ratios (i.e. fraction of experimentally observed DMRs divided by relative size of subgenomic compartment) of hyper- and hypomethylation within CpG islands (CGI), promoters that contain or do not contain CGIs, promoter-to-exon junctions, exons, introns, intergenic and repeat regions are given. Dashed line demarcates over- versus underrepresentation. d)–f) Validation of genome-wide MeDIP-seq data, using bisulfite pyrosequencing methodology d) Validation of DMR within *Ush1g* by bisulfite pyrosequencing using two samples that were subjected to MeDIP-seq and nine additional samples. Percent Methylation of all CpGs across the complete regions is given, colour code as in b). e) High-resolution graphical reconstruction of bisulfite pyrosequencing results for *Ush1g* DMR region 1, samples B3, N5, Ad5. Red: Methylated; blue: Unmethylated CpG f) Comparison of MeDIP-seq and bisulfite pyrosequencing data, as shown in Table S4c. y-axis represents MeDIP-seq derived and MEDIPS normalized rms-values (log2 scale) for cross-validated genomic regions from three samples (one sample each B6, APC^Min^ normal, APC^Min^ adenoma). Box plots depict MeDIP rms values for different methylation classes, as defined by bisulfite pyrosequencing. It is of note that MeDIP-seq procedures cannot detect DMRs with constant reliability over the complete genome, and may under-represent repetitive regions and regions with low CpG density.

To investigate DNA methylation patterns that occur shortly after intestinal tumour initiation in the mouse, we compared normal and adenomatous tissues of wildtype C57BL/6J (B6) and isogenic APC^Min^ mice by immunoprecipitation of methylated DNA followed by massively parallel sequencing (MeDIP-seq) [6], [7], [26]. Three intestinal samples of normal B6 mice, as well as three normal intestinal and five adenoma samples of B6-APC^Min^ mice were subjected to MeDIP-seq to generate a total of 5.6×10^8^ 36mer reads, which were mapped to the mouse genome [27] (approximately 2×10^7^ uniquely mapped reads per sample; see Figure 1a for summary of samples, and Table S1 for read generation and mapping statistics). To test for efficacy of the immunoprecipitation, we analysed a highly methylated and a CpG-free control region by quantitative PCR (*Xist*, Csa; [6]), and found a median enrichment of 72-fold (Figure S1). Pairwise Pearson's correlations revealed a high overall similarity between the MeDIP-seq samples (r = 0.88 to 0.93; Table S2).

To identify focal methylation differences between normal tissue and adenoma, we calculated read density in overlapping 500 bp windows over the complete genome, and compared the six normal with the five adenoma samples (Wilcoxon P<0.01, mean signal in at least one group >0.25 mapped reads per million, ratio between normal and adenoma >1.33). We identified 13,980 DMRs, of which 5,135 (37%) were hypermethylated and 8,845 (63%) hypomethylated in adenoma (see Figure 1b for a characteristic hypermethylated DMR within *Ush1g*; Table 1 for DMR summaries; Table S3 for a full list of DMRs).

**Table 1:** Numbers of DMRs identified by MeDIP-seq, and assignment to subgenomic regions.

DMR	total	CGI	Pro	CGI-Pro	Exon	Exon-Pro	Intron	Inter-genic	Repeat	Vert. Cons.
**Hyper**	5135	542	582	65	1113	293	2865	2060	3517	2120
**Hypo**	8845	611	723	105	1141	317	4792	3875	3875	3465
**Total**	13980	1153	1305	170	2254	610	7657	5935	7392	5585

In total 6123 500 bp-windows met criteria for hypermethylation in adenoma and resulted in 5135 merged hypermethylated DMRs; 11567 500 bp-windows were identified as hypomethylated in adenoma and resulted in 8845 merged hypomethylated DMRs; on average, hypomethylated DMRs were larger compared to hypermethylated regions.

CGI, CpG island; Pro, Promoter (−1 kb to +0.5 kb of the TSS); CGI-Pro, Promoter containing CpG island, Exon-Pro, Promoter-to-exon junction; Vert. Cons., conserved vertebrate elements. Repeats were identified using the Repeat Masker table and conserved elements using the conserved Vertebrate phastconsElements30way table provided by UCSC [60].

Using odds ratio calculation, we found that methylation changes were unevenly distributed over the genome. Both hyper- and hypomethylated DMRs were found highly enriched in CpG islands, promoter and exon regions. In contrast, lower frequencies of DMRs were observed in repetitive, intergenic and intronic regions (Figure 1c). In particular, we did not observe general hypomethylation in Line-1 and IAP-repeats, which is in contrast to advanced human colon carcinomas that frequently show defects in the maintenance of repeat methylation [11], [28], [29] (Table S4a). It is however of note that the highest absolute numbers of DMR windows were detected in repetitive, intergenic and intronic regions, which make up the largest fraction of the genome (Table 1, Figure S2). Methylation patterns present at DMRs clearly separated normal intestine from adenoma in hierarchical cluster analyses (Figure S3)

We used two bisulfite-based approaches to validate our genome-wide MeDIP data. Methylation-specific single-nucleotide primer extension followed by HPLC separation (SIRPH, [30]) on seven samples previously used in MeDIP-seq confirmed 24 of 24 tested DMRs, and was consistent with MeDIP-seq data (Median Pearson correlation of r = 0.86; Table S4b). Massively parallel bisulfite pyrosequencing provided high-resolution profiles of individual regions and validated 21 of 21 DMRs on three samples used in the genome-wide study (Pearson correlations of the log2 ratios r = 0.8 to 0.9; Figure 1d–1f, Table S4c, Figure 1d, 1e). Additional bisulfite analysis of nine independent samples that were not used for MeDIP-seq analysis confirmed 18 of the 21 DMRs (Table S4a, cut-off P<0.05; FC>1.33), while three regions displayed variations between individual mice.

We were able to exclude gross copy number changes, which might have occurred in adenoma, by low-coverage genomic DNA sequencing of two normal intestinal samples and one adenoma (Table S1). Overall, these multiple tests validate our genome-wide DMR maps for intestinal adenoma.

### DNA hypermethylation in adenomas prevails at Polycomb target genes

**Figure 2:**
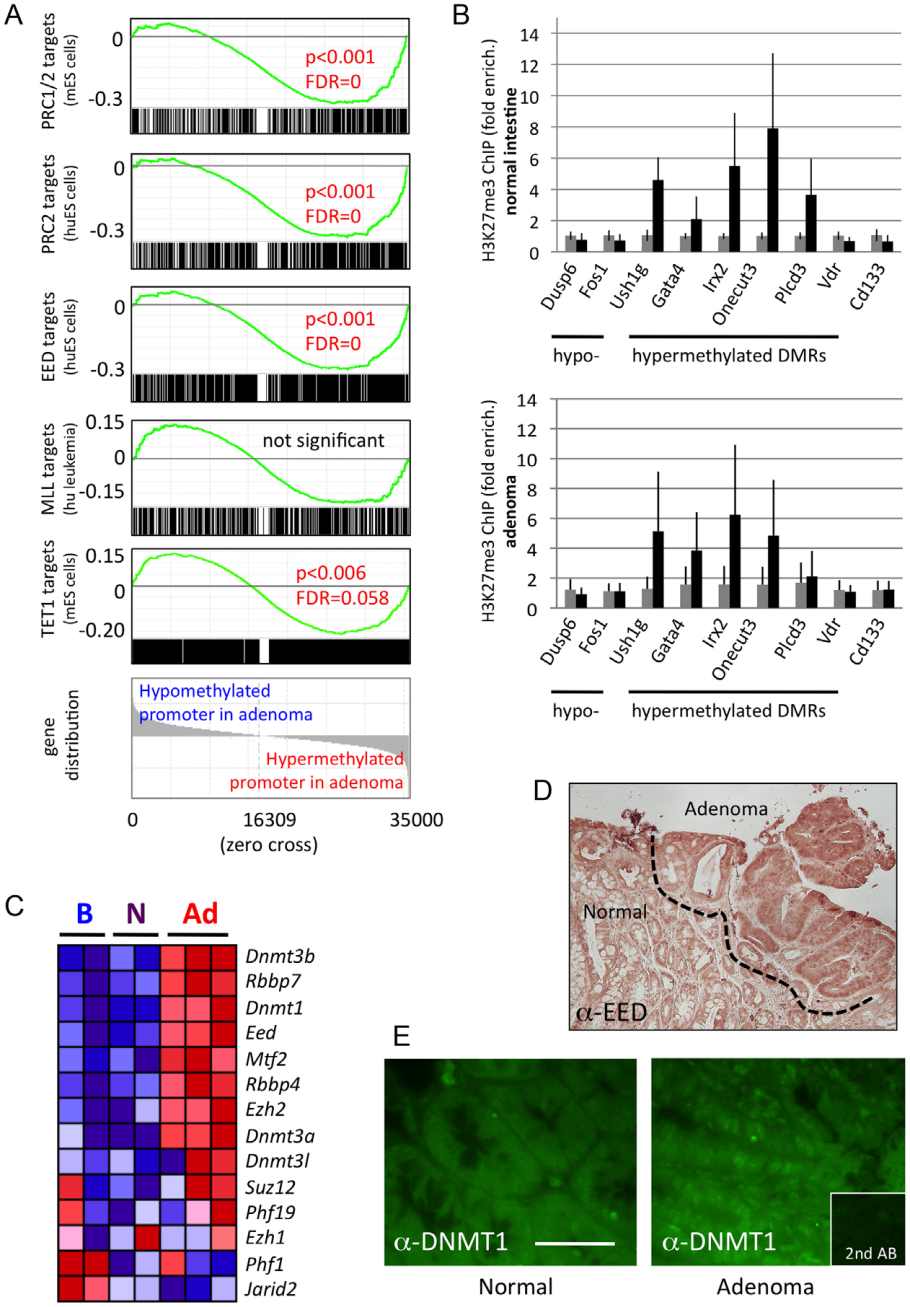
Hypermethylated DMRs are associated with Polycomb targets. a) Gene Set Enrichment Analysis (GSEA) [35] is used to probe established epigenetic signatures. Mouse genes were ordered by normal (6 samples) versus adenoma (5 samples) promoter methylation (−1,0 to +0,5 kb). Gene signatures comprising PRC1/2 target genes or mouse homologues of human targets of PRC2 complexes, EED targets, MLL targets or TET1 targets were mapped onto the ordered list, and enrichment at the extremes (hypo- or hypermethylation) was assessed. PRC and EED targets were found strongly enriched among hypermethylated promoters, while no enrichment was detected for MLL targets. TET1 targets were found weakly enriched among hypermethylated promoters, probably due to their known association with PRC complexes, and prevalence at CpG-rich sites [34]. Enrichment score graphs (top, green), signature gene distributions (black line graphs, below ES curves), p-values and false discovery rates (FDRs) are given. Significance cut-offs were P<0.05, FDR<0.25. b) Analysis of H3K27me3 marks in chromatin of mouse intestinal epithelium (n = 4 biological replicates) and adenoma (n = 3), using chromatin immunoprecipitation, followed by qPCR. black bars: Immunoprecipitated chromatin, grey: Input chromatin. Error bars give standard deviation. c) Expression of genes coding for PRC2 components or DNA methyl transferases, as determined by RNA-seq. Gene expression is colour-coded: red, high relative expression; blue, low relative expression. d) Immunohistochemical staining of EED in mouse intestine and adenoma. Dotted line demarcates normal intestinal tissue from adenoma. Adenoma contains higher levels of cytoplasmic and nuclear EED protein. e) Immunofluorescence analysis of DNMT1 in a section of normal intestine and adjacent adenoma of the mouse. Adenoma displays distinct nuclear fluorescence for DNMT1. Scale bar is 50 µm.

It is well established that DNA methylation of gene control regions is accompanied by histone modification. Since we found an enrichment of DMRs in promoters and gene bodies, we examined if adenoma methylation patterns correlated with known Polycomb Repressive Complex (PRC), Trithorax Group (TrxG/MLL) or TET1 binding sites [31]–[34]. Using Gene Set Enrichment Analysis (GSEA [35]), we found that genes previously identified as PRC1/2 targets in mouse embryonic stem cells were enriched among the genes whose promoters were hypermethylated in adenoma (Figure 2a, see Table S5 for gene-centric MeDIP-seq data, and Table S6 for signature genes). A similar correlation was found with mouse orthologues of PRC2 target genes identified in human ES cells, or with target genes of the PRC2 component EED. When we evaluated target genes of TrxG/MLL, no significant correlation with adenoma-specific hypo- or hypermethylation was found (Figure 2a). TET1 complexes have previously been implicated in DNA hypomethylation in tumours [17], however we found no correlation with the occurrence of hypomethylation of promoters in adenoma. These analyses indicate that PRC2 target genes are preferred targets of DNA hypermethylation in mouse intestinal adenoma.

To test directly if hypermethylated DMRs found in adenoma are enriched for the H3K27me3 histone mark set by PRC2, we examined several DMRs in chromatin derived from mouse intestine, by chromatin immunoprecipitation, followed by qPCR. We found that five out of six adenoma-hypermethylated DMRs (associated with the *Ush1g*, *Gata4*, *Irx2*, *Onecut3* and *Plcd3* genes, but not the one associated with *Vdr*) displayed an increased H3K27me3 mark in normal intestine and, to a large extent, also in adenoma, and hence were associated with PRC2 activity in the intestine (Figure 2b, and Figure S4). In contrast, two hypomethylated DMRs (*Dusp6* and *Fos*), as well as the stem cell marker gene *Cd133* were not enriched for the H3K27me3 mark.

Next we assessed the expression of genes coding for Polycomb complex components and DNA methyltransferases. We found up-regulation of the maintenance DNA methyltransferase gene *Dnmt1*, the *de-novo* methyltransferase genes *Dnmt3a* and *Dnmt3b*, and of several PRC2 component genes in adenoma compared to normal intestinal tissue (Figure 2c, see also Table S7 for additional gene sets related to epigenetic modification). In addition, immunohistochemical analysis on sections of normal and adenoma tissue found up-regulation of EED and DNMT1 protein in adenoma (Figure 2d, 2e). Taken together, our data suggest a functional correlation between increased DNA methyltransferase and PRC2 expression and CpG hypermethylation in adenoma.

### Methylation patterns of adenoma form *de novo* after cellular transformation

**Figure 3:**
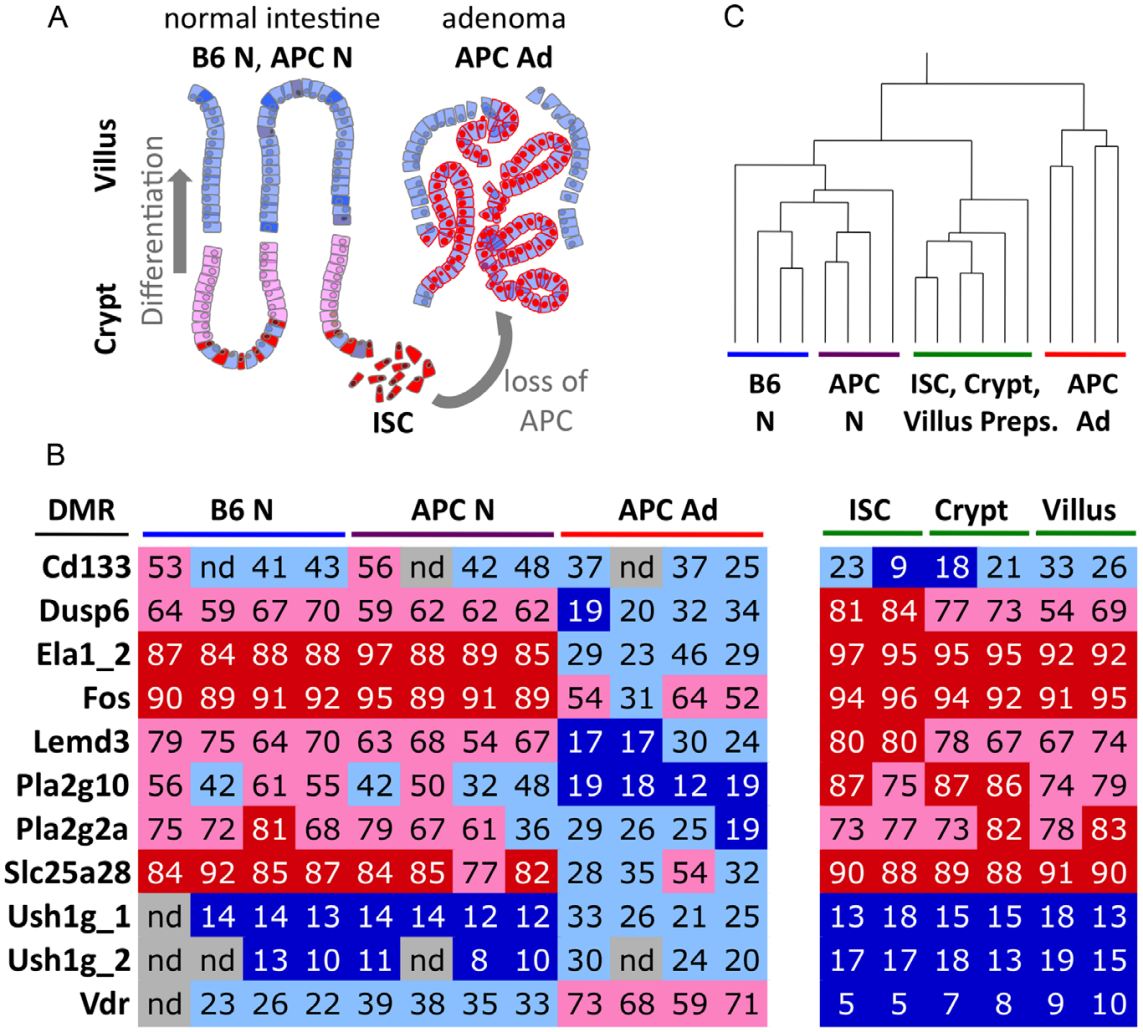
CpG methylation differentiates normal epithelial cell types from adenoma. a) Schematic representation of intestinal tissues and cell types. Differentiated villus cells are the prevailing component of bulk normal tissue samples. b) Colour-coded table of CpG methylation analyses of 11 DMRs, using bisulfite pyrosequencing. *Cd133* is an intestinal stem cell and cancer stem cell marker. *Dusp6* to *Slc25a28* represent adenoma hypomethylated DMRs, *Ush1g*_1 to *Vdr* represent adenoma hypermethylated DMRs. Percent CpG methylation within the regions are given as numbers and colour-code. Dark blue, <20% CpG methylation; light blue, 20–50% CpG methylation, light red, 50–80% CpG methylation; bright red, >80% methylation. c) Hierarchical clustering of methylation data (as shown in b) separates adenoma from normal tissue and cell preparations. Pearson correlation was employed.

The intestinal epithelium contains proliferative and differentiated cells in different domains (crypts and villi, respectively), and mouse intestinal adenoma is known to originate from intestinal stem cells (ISC) of the crypt ([36], see Figure 3a for a schematic representation of intestinal cell types). ISCs make up only a small fraction of the total tissue. Therefore we wanted to exclude that the CpG methylation signatures of proliferative or stem cells, which might have been masked by prevalent patterns of differentiated cells in normal tissue, match the signatures of adenoma. We obtained tissue samples enriched for villus or crypt cells, and in addition, purified intestinal stem cells by FACS from transgenic mice expressing the fluorescent stem cell-specific Lgr5 reporter [37]. The villus, crypt and ISC preparations were subjected to bisulfite pyrosequencing of eleven specific DMRs, among them *Dusp6* and *Fos* (containing adenoma-hypomethylated DMRs), as well as *Ush1g* and *Vdr* (containing adenoma-hypermethylated DMRs). We found minimal methylation differences between stem cells, crypt (proliferating cells) and villus (differentiated cells) at all DMRs examined, and methylation levels in all three types of cell preparations by and large matched those observed in bulk normal tissue. In contrast, the methylation levels found in adenoma were distinct (Figure 3b). Hierarchical clustering further illustrated that the DNA methylation status of the DMRs chosen is quite different in adenoma as compared to normal tissues and cell preparations (Figure 3c). Importantly, these data strongly suggest that the CpG hypo- and hypermethylation patterns of intestinal tumours do not derive from ISCs, but form *de-novo* and in a recurring manner after tumour initiation following the loss of APC.

### Differential promoter methylation and differential gene transcription in adenoma do not correlate extensively

**Figure 4:**
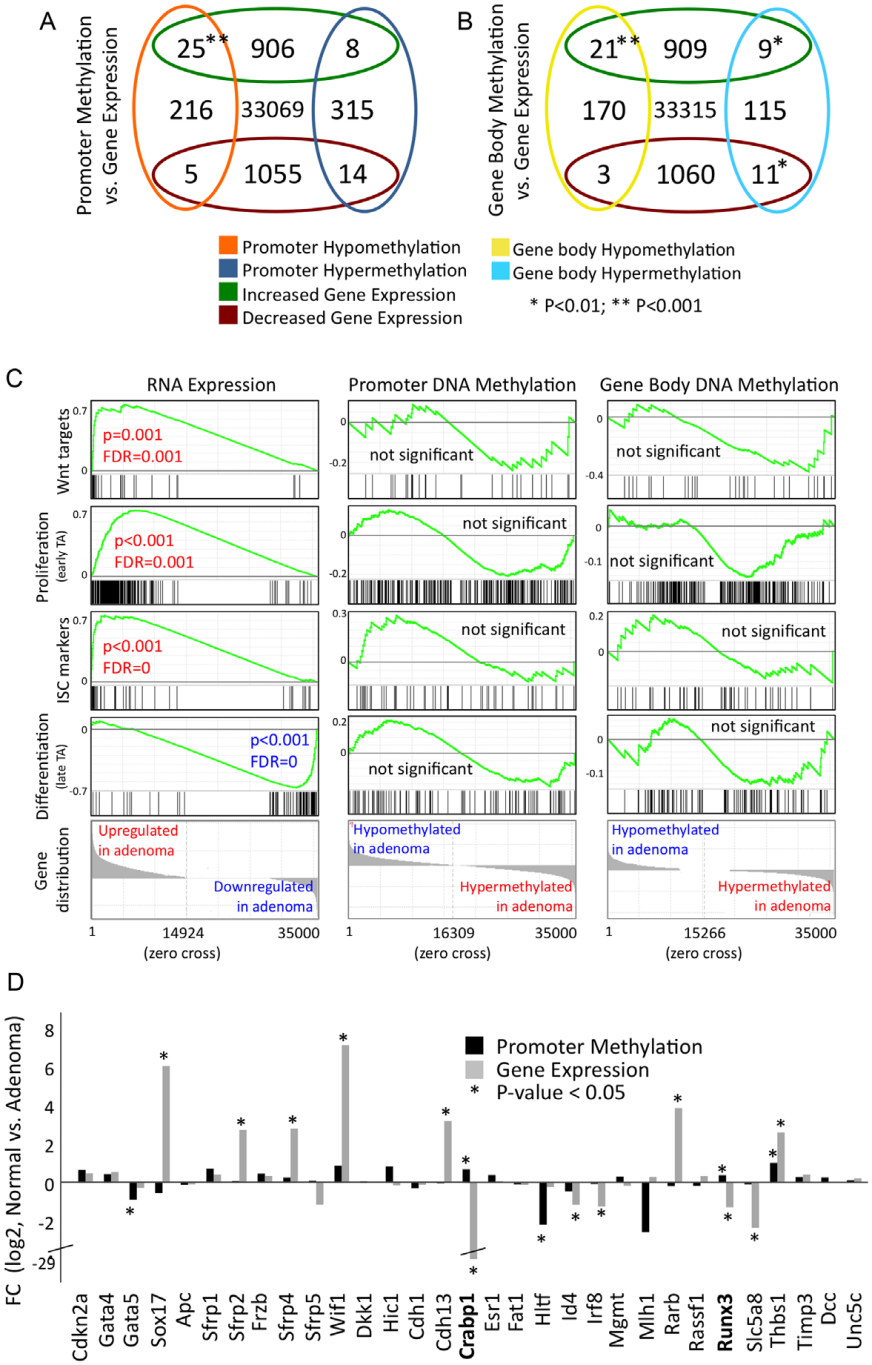
Differential gene methylation and differential gene transcription in adenoma do not correlate extensively. a)–b) Venn diagrams displaying numbers of transcriptionally regulated and differentially methylated genes. Cut-off criteria were FDR<0.001 for transcriptional regulation, and P<0.01 for methylation (calculation using edgeR). a) Genes that display differential methylation of promoter (−1.0 to +0.5 bk relative to transcription start site) b) Genes that display differential gene body methylation. c) GSEA analyses of gene signatures comprising Wnt targets, ISC-, proliferative- and differentiated cell-specific genes. Left panels: analysis of gene activity, as assessed by RNA-seq; middle and right panels: analysis of promoter or gene body methylation. Data is given as described in Figure 2a. d) Relative gene expression and promoter methylation data for 31 selected epigenetically regulated tumour suppressor genes. Fold change between normal and adenoma is given. *Crabp1* and *Runx3* are both, promoter hypermethylated and transcriptionally down-regulated. Asterisk denotes P<0.05.

It is generally assumed that promoter hypermethylation causes down-regulation of gene expression, whereas promoter hypomethylation is associated with up-regulation of genes. We asked if and to what extent this assumption holds true. To do so, we evaluated our RNA-seq data, which was derived from the same tissue samples that were used for MeDIP-seq (see Figure 1a for samples; Figure S5, S6 for RNA-seq quality assessment). We evaluated groups of genes that were both, differentially methylated and differentially expressed between normal intestine and adenoma. The number of genes that were hypomethylated at their promoters and up-regulated in adenoma was larger than expected to occur by chance (Figure 4a, see also Table S5, P<2*10^−8^, hypergeometric distribution). One example is the *Pla2g2a* gene (also known as *Mom1*), which is a known modifier of adenoma development [38] (Figure S7). Likewise, promoter hypermethylation correlated for some genes with decreased gene expression, but the overlap between promoter hypermethylated genes and transcriptionally down-regulated genes was not significant (P>0.05; Figure 4a). However, the majority of genes showed no correlation between promoter methylation and transcriptional activity, and some genes even displayed an inverse correlation. For example, *Slc9a3* contains a hypermethylated DMR in the promoter, and is strongly activated in adenoma (Figure S8). We also evaluated genes that were differentially methylated in their gene body and differentially expressed, and again found only a small overlap between changes in gene body methylation and gene expression (Figure 4b).

We asked whether gene signatures, which are representative for key cell types and processes of normal development show a common behaviour in terms of gene expression and methylation patterns in adenoma. Gene activity, as assessed by RNA-seq, differed strongly between normal intestinal tissue and adenoma for all gene signatures analysed (Figure 4c, Figure S6). We found that Wnt target genes [39], along with ISC/crypt progenitor cell and TA (transiently amplifying) cell marker genes were almost uniformly up-regulated in adenoma, while differentiation signature genes were mostly down-regulated [40]–[42] (Figure 4c, see Table S6 for signature genes). Despite the transcriptional co-regulation of the signature genes, we found no consistent or significant trend of differential promoter or gene body methylation within the ISC-, proliferative- and differentiation-associated gene groups (Figure 4c).

Previous research revealed a number of tumour suppressors, which are hypermethylated at their promoters and consequently silenced in carcinoma. We thus assessed the promoter methylation patterns and transcriptional activity of the mouse orthologues of 31 tumour suppressors previously reported to be frequently epigenetically silenced in human colon cancer [12], [24], [25]. In mouse intestinal adenoma, however, we found only two out of 31 tumour suppressors that were both, promoter-hypermethylated and transcriptionally silenced (Figure 4d). These are the retinoic-acid signal transducer *Crabp1* and the runt-related transcription factor *Runx3*, which both control differentiation and apoptosis in gut epithelia [43]–[45].

The combined data show that the correlation of differential promoter methylation and differential gene expression in mouse intestinal adenoma applies only to a small subset of genes, while most genes show an uncoupling of both processes. In particular, epigenetic silencing of tumour suppressors, which is a frequent trait in human colon carcinoma, was found to occur rarely in mouse adenoma.

### A core set of APC^Min^ adenoma-specific methylation patterns is conserved in advanced human colon cancer

**Figure 5:**
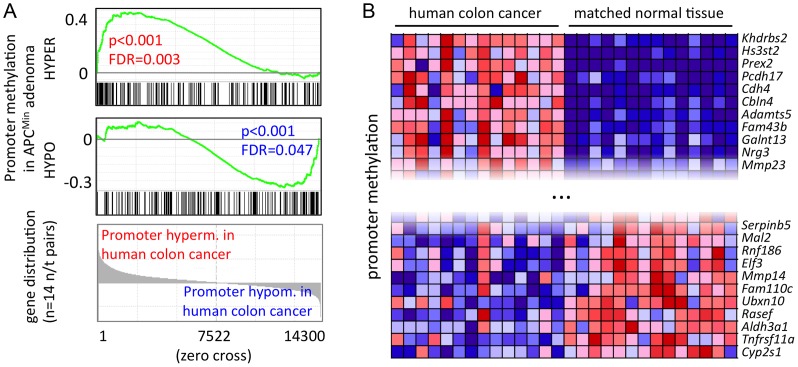
A core set of APC^Min^ adenoma-specific CpG methylation patterns is conserved in human colon cancer. a) GSEA identifies methylation changes of mouse adenoma in human colon cancer. Gene signatures comprise genes with promoter hypo- or hypermethylation in mouse adenoma (see also Figure 4a, Table S5), genes were ordered by directional methylation changes in human colon cancer (normal tissue versus carcinoma). Mouse and human gene homologues were matched using ENSEMBL Biomart (approx. 14300 unique orthologue pairs were identified). b) Promoter hypo- and hypermethlyation is conserved between mouse APC^Min^ adenoma and human colon cancer. Genes were selected from those that are significantly hyper- or hypomethylated in APC^Min^ adenoma. Conserved genes were identified as the core enrichment group of GSEA analysis in a). Figure shows top eleven hypo- and hypermethylated genes in human colon cancer. blue: low relative methylation; red: high relative methylation.

Mouse intestinal adenoma shares key functional traits and transcriptional programmes with human colon cancer [23], [46]. We therefore asked whether the similarities extend to epigenetic regulation, and consequently, whether the methylation signature of mouse intestinal adenoma could be detected in human colon cancer. To this end, we identified the human orthologues of genes whose promoters are hypo- or hypermethylated in mouse adenoma, and assessed promoter methylation patterns from MeDIP-seq data of 14 advanced human colon cancers of the stages T2–T4 (unpublished data, C. G., M. R. S. *et al.*). Strikingly, we found that a significant fraction of hyper- or hypomethylated genes identified in mouse adenoma displayed matching promoter hyper- or hypomethylation in human colon cancers (Figure 5a). Among the group of the most consistently hypermethylated genes in both human colon cancer and mouse intestinal adenoma (48 genes), we found *Cdh4*, *Crabp1*, *Crmp1*, *Dbc1*, *Duox1*, *Grm7*, *Hand1*, *Hs3st2*, *Pcdh17* and *Pdpn*, which have previously been suggested as cancer biomarkers [15], [47]–[54]. Among the genes, which are consistently promoter-hypomethylated in both mice and humans (54 genes), we identified the matrix metalloproteinase and colon cancer prognostic marker *Mmp14*, which is also over-expressed in mouse adenoma. Promoter methylation changes in the genes identified here were quite consistent across the panel of the 14 human colon cancers (see Figure 5b for a selection of top eleven hypo- and hypermethylated genes, and Table S8 for complete gene sets).

Our data define a core methylation signature of intestinal cancer, which is conserved between humans and mice. The comparison of mouse with human data (comprising early lesions in mice and advanced stages of human cancer) indicates that this core epigenetic signature is established early during tumour formation, and is retained when the cancer epigenome is further modified during tumour progression to carcinoma.

## Discussion

We report that the formation of APC^Min^ mouse adenoma from normal intestinal tissue is accompanied by characteristic CpG methylation changes in a large number of genomic regions, and that a core set of such DMRs is conserved between mouse adenoma and human colon cancer. The finding of recurring DMRs in independent adenomas is in agreement with an instructive mechanism guiding CpG methylation in tumours, as proposed previously [7], [15]. It has been suggested that gain of CpG methylation in tumour cells correlates with the H3K27me3 mark set by PRC2 activity [15], [55], and our data demonstrating preferential DNA hypermethylation of Polycomb target sites confirm this model. Furthermore, our gene expression data show that PRC2 components are over-expressed in adenoma, and immunohistochemistry confirmed up-regulation of EED protein in adenoma tissue. These observations suggest that enhanced PRC2 activity may be instrumental in the rapid establishment of the adenoma-specific DNA hypermethylation pattern following the loss of APC. Hypomethylation in mouse adenoma was likewise found to occur in a regular pattern, rather than in the form of genome-wide demethylation. We could however not identify correlations between recurring hypomethylation and binding patterns of epigenetic regulators, such as PRC, TrxG or TET complexes.

**Figure 6:**
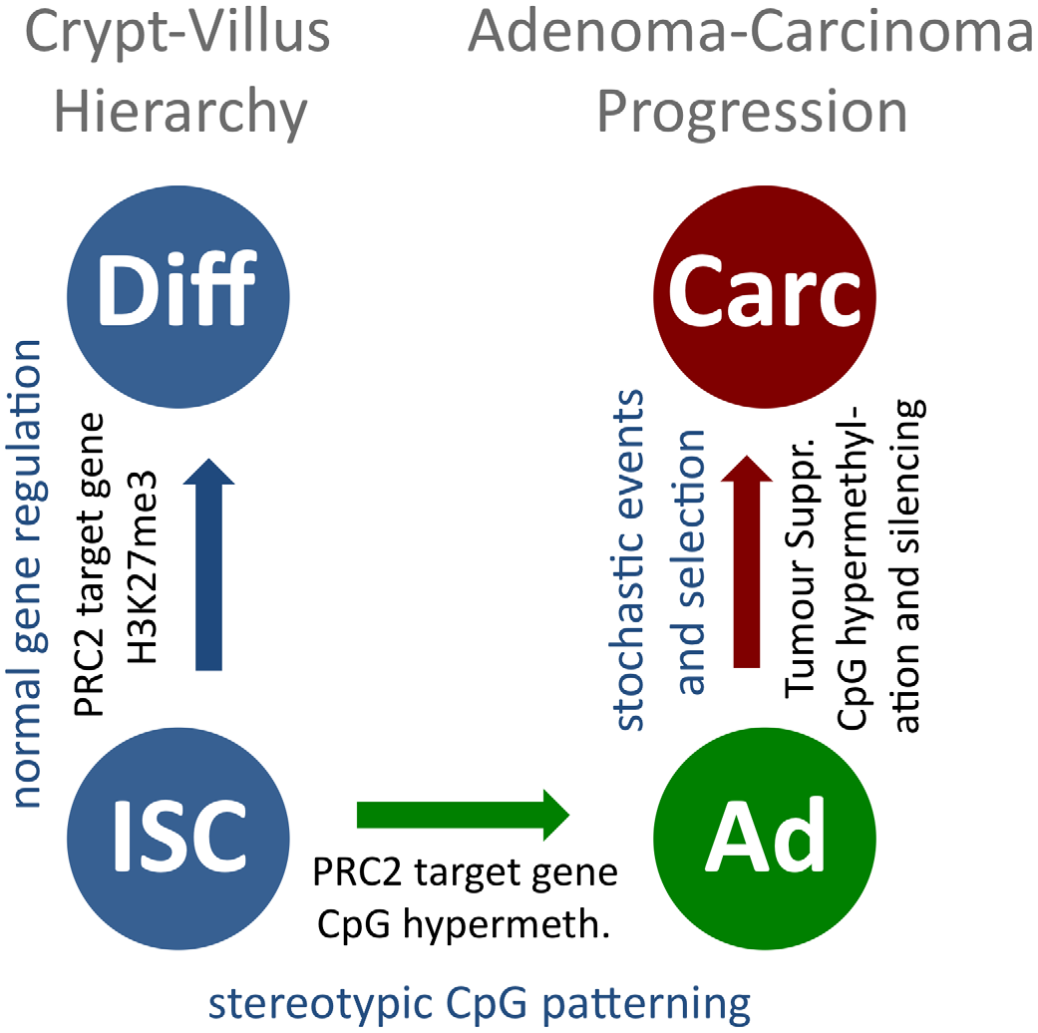
A model for stepwise formation of cancer cell CpG epigenomes. CpG methylation is uniform within the normal cellular hierarchy of the intestine, and PRC2-associated H3K27me3 marks are present in crypt and villus cells (blue, to the left). Upon tumour initiation, recurring CpG methylation patterns form, guided by an instructive mechanism that is linked to PRC2 for hypermethylated sites (blue to green). Further CpG methylation changes occur slowly, probably in a stochastic manner. A fraction of these bestow tumour cells with a selective advantage and are subject to clonal expansion during tumour progression (green to red).

Previous reports have shown that in human colon cancer tumour suppressors are frequently silenced by promoter methylation, and individual tumours differ in the genes that are silenced [12], [24], [25]. In contrast, we rarely detected tumour suppressors among the recurring DMRs, which are common between individual APC^Min^ adenomas and partly shared between mouse adenoma and human colon cancer. These observations suggest that the variable epigenetic patterns of individual human colon cancers derive by clonal expansion following stochastic methylation changes that confer a growth advantage to individual tumour cells. Since such clonal selection is dependent on rare initiating events, tumour suppressor silencing is not detected in early APC^Min^ adenoma, but frequently and variably with respect to the genes affected in human colon cancer. This different situation also applies to genomic instability [56]. It thus appears likely that most epigenetic changes in tumour suppressor genes represent late steps that arise during adenoma-to-carcinoma progression rather than representing differences between the mouse and human species. Taken together, our combined analyses suggest that the epigenetic deregulation in tumours can be divided into two principal components, an early and fast instructive patterning mechanism, which immediately follows tumour initiation, and late rare stochastic events promoting clonal expansion (for a model, see Figure 6).

We present for the first time DNA methylation data comparing primary normal cell types with transformed tissue derived from the same organ. We observed that the adenoma-specific DNA methylation patterns were distinct from those found in the major cell types of intestinal epithelia, including intestinal stem cells, which give rise to adenoma in the mouse [36]. This indicates that adenoma-specific DMRs are not pre-existing in a cell population comprising a minor fraction of the intestinal tissue, such as ISCs, and simply emerge by expansion of this cell type in adenoma, but are formed *de-novo* in a recurring and characteristic manner. Our data therefore establish beyond doubt that epigenetic patterns found in tumour cells arise upon transformation, and are thus suitable as specific epigenetic disease markers.

Current epigenomic models assume that DNA hypermethylation, in particular methylation of promoter regions, means gene silencing. We find that this model, with respect to adenoma, holds true only for a minor fraction of genes whose promoters are hypermethylated. Likewise, only a minor fraction of genes with hypomethylated promoters in adenoma are transcriptionally up-regulated. These gene groups are larger than expected to occur by chance, however, the large majority of genes does not follow this simple logic. Overall, changes in promoter methylation do not appear to generally cause altered gene expression. Further studies involving additional control elements such as enhancers and intragenic silencers are required for a deeper understanding of the control of gene expression by epigenetic mechanisms in adenoma.

We identified a core set of recurring DMRs, which is conserved between mouse adenoma and human colon carcinoma. This has important clinical implications, since our finding suggests the existence of a conserved set of DMRs arising early in human colon cancer, which may be universal to a large majority of human intestinal malignancies. This class of biomarkers may be suitable for the discovery of intestinal cancer at an early stage.

We rarely found hypermethylation and silencing of specific tumour suppressors in early adenoma of the mouse. Epigenetic silencing of tumour suppressors by promoter hypermethylation is however frequent in a subset of advanced human tumours. These latter marks may be useful as markers, which may correlate with histopathological features and be suited for distinguishing clinically relevant tumour subtypes.

## Materials and Methods

### Ethics statement and mouse strains

All mouse work has been conducted according to the relevant national and international guidelines. APC^Min^ mice (genetic background C57BL/6J (B6), Jackson laboratory (www.jax.org, USA)), were maintained by backcrossing to C57BL/6JOlaHsd (Harlan, The Netherlands). Animals were housed at a 12 h/12 h light/dark cycle and fed *ad libitum*. B6-APC^Min/+^ (APC^Min^) and B6-APC^+/+^ (B6) wild type littermates were dissected at the age of 15 to 19 weeks and individual adenomas and normal intestinal tissue was excised from the ileum.

### Histology and cell-based methods

For immunohistochemistry, tissues were fixed in 4% formaldehyde, dehydrated via a graded ethanol series, embedded in paraffin, and sectioned at 4 µm. Anti-EED (1∶50, ARP-38384 Aviva) and anti-DNMT1 (1∶200, Alexis 804-369-C100) primary antibodies, anti-mouse Alexa488 and ImmPRESS secondary antibodies, and the NovaRED substrate kit (Vector) were used. Crypt, villus and intestinal stem cell preparations were prepared as described previously [57]. In short, villus preparations were scraped from the inner intestinal surface after initial washing steps, and crypts were isolated by filtering (70 µm, CellTRICS) after 30 min of PBS/2 mM EDTA incubation. FACS sorting for GFP^+^ intestinal stem cells was done after TrypLE dissociation of cells, using a FACS Aria system (BD) and a 100 µm nozzle. Crypt and villus tissue preparations were controlled for enrichment by visual inspection.

### DNA and RNA methods

In short, DNA and RNA of normal and adenoma intestinal samples were isolated using the Allprep DNA/RNA Mini kit (Qiagen). A DNAse digest of the RNA was performed on the columns according to the manufacturer's instructions. Nucleic acid concentrations were measured with a Nanodrop photometer (Implen, Germany) or Qubit fluorometer (Invitrogen) and quality was assessed on an agarose gel (DNA) or a 2100 Bioanalyzer (Agilent, RNA). MeDIP-seq was adapted from previously published protocols [6], [26], [58]. RNA-seq was performed from 4 µg of total RNA, after depletion for ribosomal RNA using the RiboMinus Eukaryote Kit for RNA-seq (Invitrogen), following a strand-specific protocol [59]. Sequencing was done on 454 GS-FLX for the bisulfite-pyrosequencing and Illumina Genome Analyser (GAIIx) machines for MeDIP-seq and RNA-seq. Extended RNA/DNA material and methods are available online as Text S1.

### Bioinformatic analysis

Bioinformatic methods section is available online as Text S1 along with associated Tables S9, S10. MeDIP-seq and RNA-seq data can be accessed in GEO under GSE38983.

## Supporting Information

Figure S1 MeDIP quality controls. a) Enrichment of methylated DNA in the MeDIP samples, as assessed by qPCR of a methylated (*Xist*) and a CpG free control region (Csa, [6]) for the 11 samples. Shown is the fold change (2^−ΔCT^). Data for individual samples are given in Table S1. b) Relative frequency of CpG enrichment, as calculated by MEDIPS (Chavez et al., 2010) for the 11 MeDIP and the 3 input samples. c) Fractions of uniquely aligned reads for the 11 MeDIP and the 3 input samples. Input samples display a higher percentage of uniquely aligned reads, most likely due to immunoprecipitation of methylated repetitive regions. d, e) Comparison of MeDIP-seq data with BS-pyrosequencing. Three samples (representing the B, N, Ad groups) were used for comparative analyses. d) Shown are the log_2_ values of the % methylation as determined by BS-pyrosequencing on the x-axis and the log_2_ values of the MeDIP-seq rpm value on the y-axis. The three MeDIP-seq rpm values of 0 were transformed to 0.01 in order to calculate a log_2_ value. Pearson's correlation is r = 0.85. e) The log_2_ values of the MeDIP-seq rms values normalized for CpG content are given on the y-axis. The three MeDIP-seq rms values of 0 were transformed to 0.001 in order to calculate a log_2_ value. Pearson's correlation is 0.89. Data are given in Table S4c.
(TIF)

Figure S2 Assignment of DMRs to genomic features. a) Assignment of DMRs to genomic features, as given in Table 1. b) Assignment of DMRs to repetitive elements. c) Numbers and percentage of DMRs localized to repetitive elements. The full list of DMRS is given in Table S5. d) Odds ratios of hypermethylated and hypomethylated 500 bp windows that map to repetitive elements. Enrichment and depletion are given relative to all hyper- or all hypomethylated 500 bp windows. A slight enrichment of hypomethylated regions was observed for the LINE-L2 and SINE elements, whereas a depletion of differentially methylated 500 bp windows was observed in LTR and LINE-L1 elements. In contrast, when assessing the methylation in LINE-L1 and IAP elements by BS-seq in normal intestine and adenoma, no methylation differences were observed (Table S4a). MeDIP analyses take into account only uniquely aligned reads, which may interfere with analyses of highly repetitive sequences.
(TIF)

Figure S3 Clustering of genome-wide methylation profiles. a)–d) Unsupervised clustering of genome-wide MeDIP profiles (3× B6 normal intestine, 3× APC^Min^ normal intestine, 5× adenoma), using 500 bp windows covering the genome. Windows were filtered for read density (>0.25 rpm, i.e. omitting genomic regions with no or few mapped reads) and certain thresholds for the coefficient of variance (cv) were applied, i.e. clustering was stepwisely restricted to a smaller, but more methylation-variable fraction of the genome, as indicated. These unsupervised clustering variants do not fully separate normal and tumour tissue. e) Clustering using the DMRs identified. As expected, a clear separation between tumour and normal tissue is observed. f) Unsupervised clustering using promoter regions. Promoters were filtered for read density and variation (>0.1 rpm; cv>0.5). This clustering approach does not fully separate normal and tumour tissue. For sample details, see Figure 1a.
(TIF)

Figure S4 Histone H3K27me3 methylation of DMRs in purified crypt or villus cells. Chromatin was isolated from mouse intestinal crypts (above) or villi (below), and analysed using H3K27me3 chromatin immunoprecipitation, followed by qPCR. black bars: Immunoprecipitated chromatin, grey: Input chromatin. Error bars give standard deviation in three biological replicates.
(TIF)

Figure S5 Quality assessment of the RNA-seq data a) RNA-seq read distribution, as percentage of all generated reads. b) Dendrogram showing the hierarchical clustering of the RNA-seq data. c) Spearman's correlation of the RNA-seq samples, as underlying the dendrogram shown in b). The correlations were calculated using those ENSEMBL genes with at least 20 exon read counts in at least one of the samples. rho>0.9 is displayed in grey. The normal intestinal samples of both, Apc^Min^ (N) and B6 (B) are very similar, while most changes in expression are found in adenoma (Ad) d) Validation of the RNA-seq data, by qRT-PCR. Shown are the log_2_ ratios of the RNA-seq data on the y-axis and the log_2_ ratios of the qPCR on the x-axis. For the qPCR the same samples that were used for RNA-seq and in addition, four independent samples per group were used. RNA-seq and qRT-PCR are in good agreement. The oligos used for the RT-qPCR experiments are given in Table S10.
(TIF)

Figure S6 Comparative and KEGG pathway analysis of RNA-seq data. a,b) Venn diagrams display the intersection of differentially expressed genes as determined by edgeR (FDR<0.001) for genes a) up-regulated in adenoma compared to normal intestinal tissue of B6 or APC^Min^ (Ad vs. B, Ad vs. N) and normal intestinal tissue of APC^Min^ compared to normal intestinal tissue of B6 (N vs. B); b) down-regulated in adenoma compared to normal intestinal tissue of B6 or APC^Min^ (N vs. Ad, B vs. Ad) and down-regulated in APC^Min^ normal intestinal tissue compared to B6 (B vs. N). Analyses demonstrate similarity between the normal (B, N) samples, while adenoma (Ad) differs. c,d) KEGG pathway analyses. Overrepresented KEGG pathways in genes up-regulated (c) and down-regulated (d) in adenoma compared to normal intestinal tissue of both, APC^Min^ and B6 as caculated by edgeR (FDR<0.001) are shown. The x-axis displays the −log10 of the p-value calculated by DAVID (http://david.abcc.ncifcrf.gov). Cancer-related pathways are up-regulated in adenoma, while pathways related to immune function are down-regulated. B, normal intestinal tissue from B6; Ad, adenoma from APC^Min^; N, normal intestinal tissue from APC^Min^.
(TIF)

Figure S7 Association of hypomethylation and transcriptional up-regulation in the *Pla2g2a* locus. a) UCSC browser track of the MeDIP-seq data. The black track displays the identified adenoma-hypomethylated DMR and the position of the validated region. Colour code is as in Figure 1b. b) Heat map of the 454 GS-FLX-based bisulfite pyrosequencing for the *Pla2g2a* region shown in a. Samples B3, N5 and Ad5 are given. Each column represents a CpG, and each row a generated sequencing read. Red: methylated CpG, blue: unmethylated CpG, white: no data. c) Expression for *Pla2g2a* and the neighbouring *Pla2g5* gene, as determined by RNA-seq. Expression is given as the log_2_ fold change, as calculated by edgeR for the comparison adenoma vs normal intestinal samples. Both genes are significantly deregulated (FDR<0.001). The elevated expression of *Pla2g2a* was validated by qRT-PCR using additional samples (data not shown). *Pla2g2a* is also known as *Mom1* (Modifier of intestinal neoplasia 1).
(TIF)

Figure S8 Association of hypermethylation in the Slc9a3 and the *Ush1g* regions with transcriptional up-regulation. a, b) Promoter hypermethylation in *Slc9a3* is associated with transcriptional activation. a) UCSC browser track of MeDIP-seq data. Colour code is as in Figure 1b. b) Expression for *Slc9a3* as determined by RNA-seq. Expression is given as the log_2_ fold change, as calculated by edgeR for the comparison adenoma versus normal intestinal samples. c,d) Methylation marks in the *Ush1g* region are associated with transcriptional up-regulation of five neighbouring genes within a 90 kb region. c) UCSC browser track of the MeDIP-seq data. The track “Ad hyper” depicts hypermethylated and “Ad hypo” hypomethylated DMRs. Light red overlay: position of a adenoma-hypermethylated DMR, as identified by MeDIP-seq and validated by BS-pyrosequencing; light green overlay: position of a hypermethylated and a hypomethylated DMR next to each other. d) Expression of six adjacent genes (*Fads6*, *Otop2*, *Ush1g*, *Otop3*, *C630004H02Rik* and *Cdr2l*), as determined by RNA-seq. Expression is given as the log2 fold change, as calculated by edgeR for the comparison adenoma versus normal intestinal samples. The differential expression was validated by qPCR for *Otop2*, *Ush1g*, *Otop3* and *C630004H02Rik* using additional samples (data not shown). * depicts FDR<0.0000001.
(TIF)

Table S1 Sequencing statistics. The number of generated lanes, aligned single 36mer reads, uniquely aligned reads and the CpG enrichment frequency is given. The relative frequency of CpG enrichment was calculated using the MeDIPS package (Chavez et al., 2010, Genome Res. 20(10):1441–50). The CpG enrichment evaluates the frequency of CpGs within the total number of sequenced nucleotides with respect to the frequency of CpGs within the mm9 reference genome. The column qPCR shows the fold enrichment of a normally methylated region (Xist) over an unmethylated region (Csa) [6]. The sample names are as given in Figure 1a. The numbers indicate the animals, i.e. N4, Ad4 and Ad4-1 are samples derived from the same animal. Ad4 and Ad4-1 are different adenomas isolated from the same animal. MeDIP-seq and RNA-seq were generated from material obtained from the same tissue sample (i.e. MeDIP_N4 and RNA_N4).
(XLS)

Table S2 Pearson's correlation of the MeDIP-seq data.
(XLS)

Table S3 Differentially methylated regions.
(ZIP)

Table S4 Data validation a) BS-validation using bisulfite pyrosequencing on nine independent samples, that were not used for MeDIP-seq before. b) Validation of MeDIP-seq by SIRPH. c) Correlation of MeDIP-seq with bisulfite pyrosequencing data for three samples that were used for both, MeDIP-seq and bisulfite pyrosequencing.
(XLS)

Table S5 Gene-centric RNA-seq and MeDIP-seq data.
(ZIP)

Table S6 Gene Expression Signatures, as used for GSEA analysis. Signature names, References and ENSEMBL Gene Identifiers are given.
(XLS)

Table S7 Expression of selected genes related to epigenetic regulation a) Given are the gene expression values for selected gene sets as determined by RNA-seq and calculated by edgeR. The p-value given was calculated over all genes by edgeR, and is different from P-value calculated based on individual gene expression values.
(XLS)

Table S8 Lists of genes that are promoter hypermethylated or promoter hypomethylated in both, human colon cancer and mouse adenoma. Genes were initially identified by analysis of Mouse MeDIP-data, using a combined p-value and fold change score cut-off between normal and adenoma (genes were included if p<0.05, fold change FC>1.33fold, more than 10 reads in both, normal and adenoma. See also “score” in Table S5). GSEA was performed using Human MeDIP data for sorting, and the hyper- and hypomethylated mouse gene lists as signatures (see also Table S6). Genes given in this table comprise the core enriched group in GSEA analysis.
(XLS)

Table S9 Reaction conditions and primer sequences for MeDIP validation experiments.
(XLS)

Table S10 Oligonucleotide sequences used for qPCR.
(XLS)

Text S1 Supplementary Methods.
(DOC)
