# Supplementary figures and images for: DNA–Methylome Analysis of Mouse Intestinal Adenoma Identifies a Tumour-Specific Signature That Is Partly Conserved in Human Colon Cancer
Source: PLoS Genet. 2013 Feb 7;9(2):e1003250. doi: 10.1371/journal.pgen.1003250 (PMC3567140; doi:10.1371/journal.pgen.1003250)

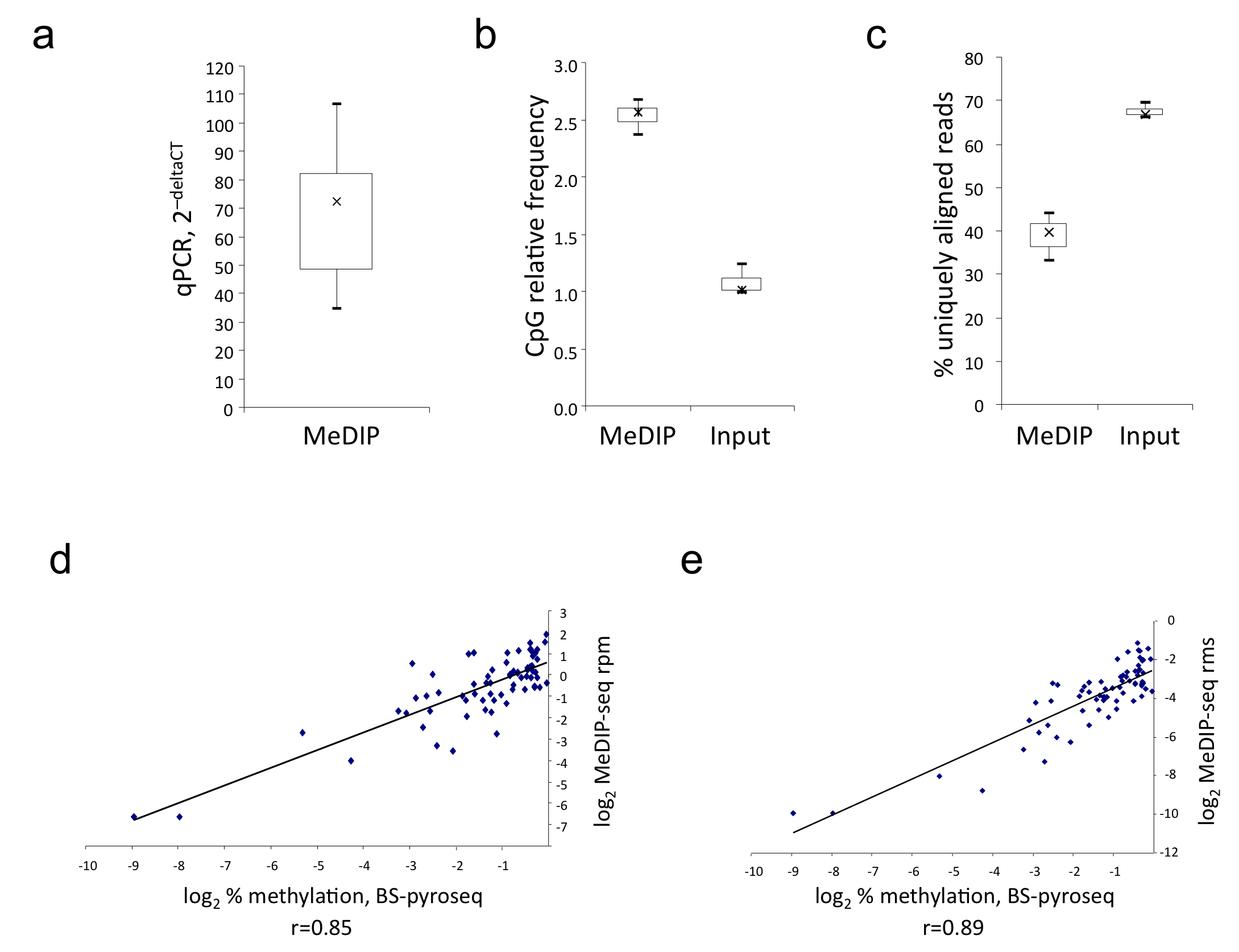

Supplement: Figure S1 — MeDIP quality controls. a) Enrichment of methylated DNA in the MeDIP samples, as assessed by qPCR of a methylated (Xist) and a CpG free control region (Csa, [6]) for the 11 samples. Shown is the fold change (2−ΔCT). Data for individual samples are given in Table S1. b) Relative frequency of CpG enrichment, as calculated by MEDIPS (Chavez et al., 2010) for the 11 MeDIP and the 3 input samples. c) Fractions of uniquely aligned reads for the 11 MeDIP and the 3 input samples. Input samples display a higher percentage of uniquely aligned reads, most likely due to immunoprecipitation of methylated repetitive regions. d, e) Comparison of MeDIP-seq data with BS-pyrosequencing. Three samples (representing the B, N, Ad groups) were used for comparative analyses. d) Shown are the log2 values of the % methylation as determined by BS-pyrosequencing on the x-axis and the log2 values of the MeDIP-seq rpm value on the y-axis. The three MeDIP-seq rpm values of 0 were transformed to 0.01 in order to calculate a log2 value. Pearson's correlation is r = 0.85. e) The log2 values of the MeDIP-seq rms values normalized for CpG content are given on the y-axis. The three MeDIP-seq rms values of 0 were transformed to 0.001 in order to calculate a log2 value. Pearson's correlation is 0.89. Data are given in Table S4c. (TIF) [file pgen.1003250.s001.tif]

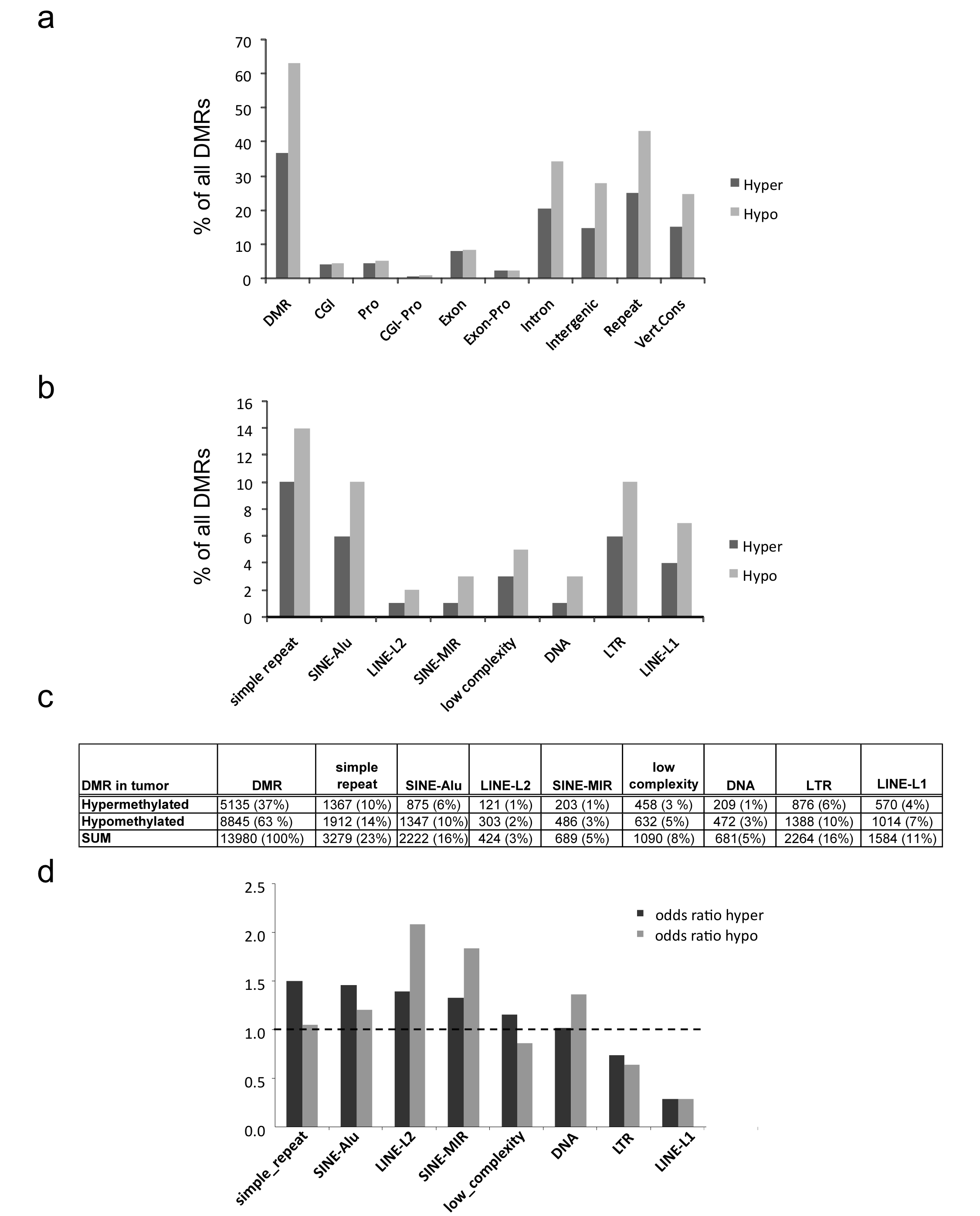

Supplement: Figure S2 — Assignment of DMRs to genomic features. a) Assignment of DMRs to genomic features, as given in Table 1. b) Assignment of DMRs to repetitive elements. c) Numbers and percentage of DMRs localized to repetitive elements. The full list of DMRS is given in Table S5. d) Odds ratios of hypermethylated and hypomethylated 500 bp windows that map to repetitive elements. Enrichment and depletion are given relative to all hyper- or all hypomethylated 500 bp windows. A slight enrichment of hypomethylated regions was observed for the LINE-L2 and SINE elements, whereas a depletion of differentially methylated 500 bp windows was observed in LTR and LINE-L1 elements. In contrast, when assessing the methylation in LINE-L1 and IAP elements by BS-seq in normal intestine and adenoma, no methylation differences were observed (Table S4a). MeDIP analyses take into account only uniquely aligned reads, which may interfere with analyses of highly repetitive sequences. (TIF) [file pgen.1003250.s002.tif]

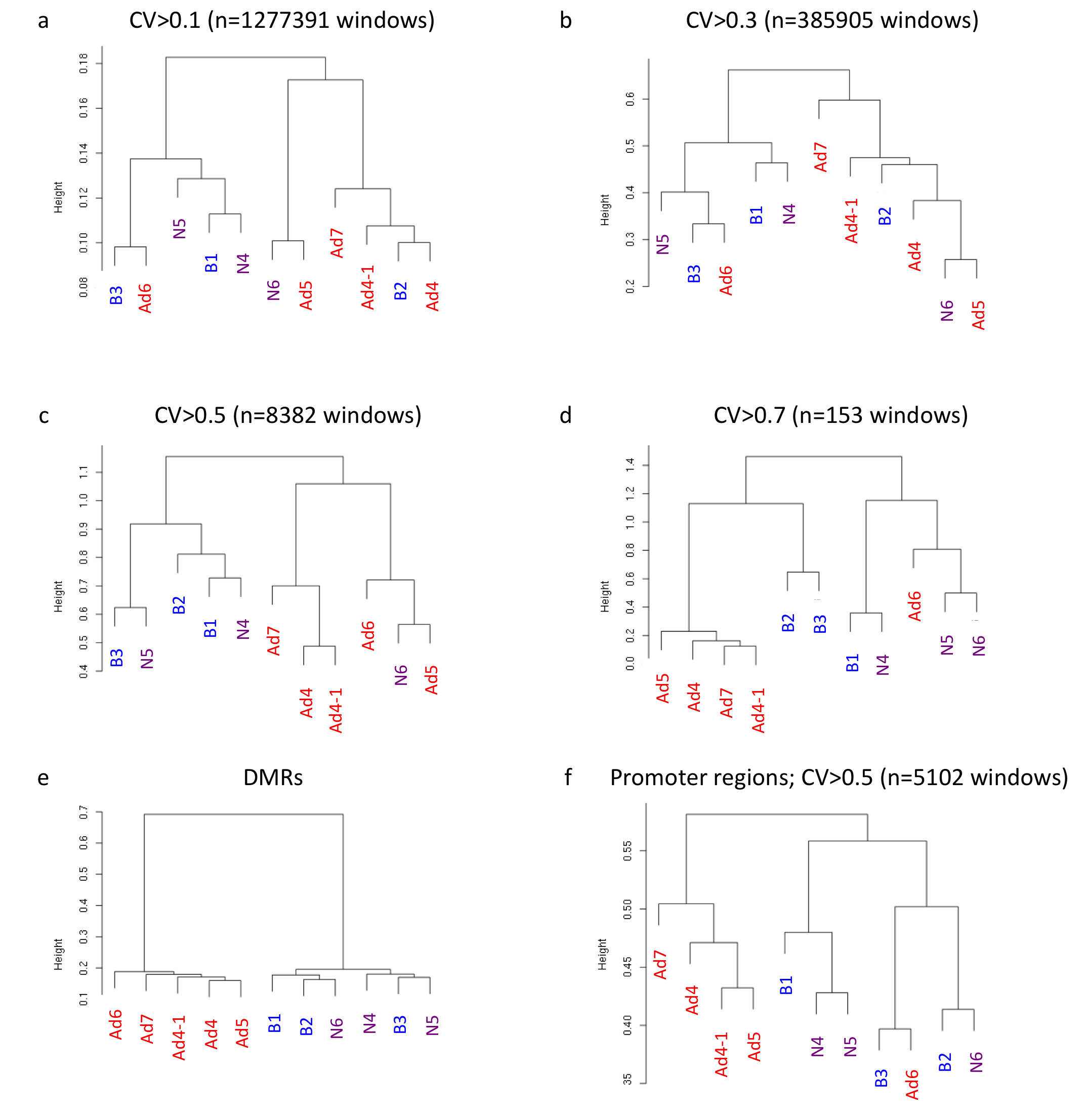

Supplement: Figure S3 — Clustering of genome-wide methylation profiles. a)–d) Unsupervised clustering of genome-wide MeDIP profiles (3× B6 normal intestine, 3× APCMin normal intestine, 5× adenoma), using 500 bp windows covering the genome. Windows were filtered for read density (>0.25 rpm, i.e. omitting genomic regions with no or few mapped reads) and certain thresholds for the coefficient of variance (cv) were applied, i.e. clustering was stepwisely restricted to a smaller, but more methylation-variable fraction of the genome, as indicated. These unsupervised clustering variants do not fully separate normal and tumour tissue. e) Clustering using the DMRs identified. As expected, a clear separation between tumour and normal tissue is observed. f) Unsupervised clustering using promoter regions. Promoters were filtered for read density and variation (>0.1 rpm; cv>0.5). This clustering approach does not fully separate normal and tumour tissue. For sample details, see Figure 1a. (TIF) [file pgen.1003250.s003.tif]

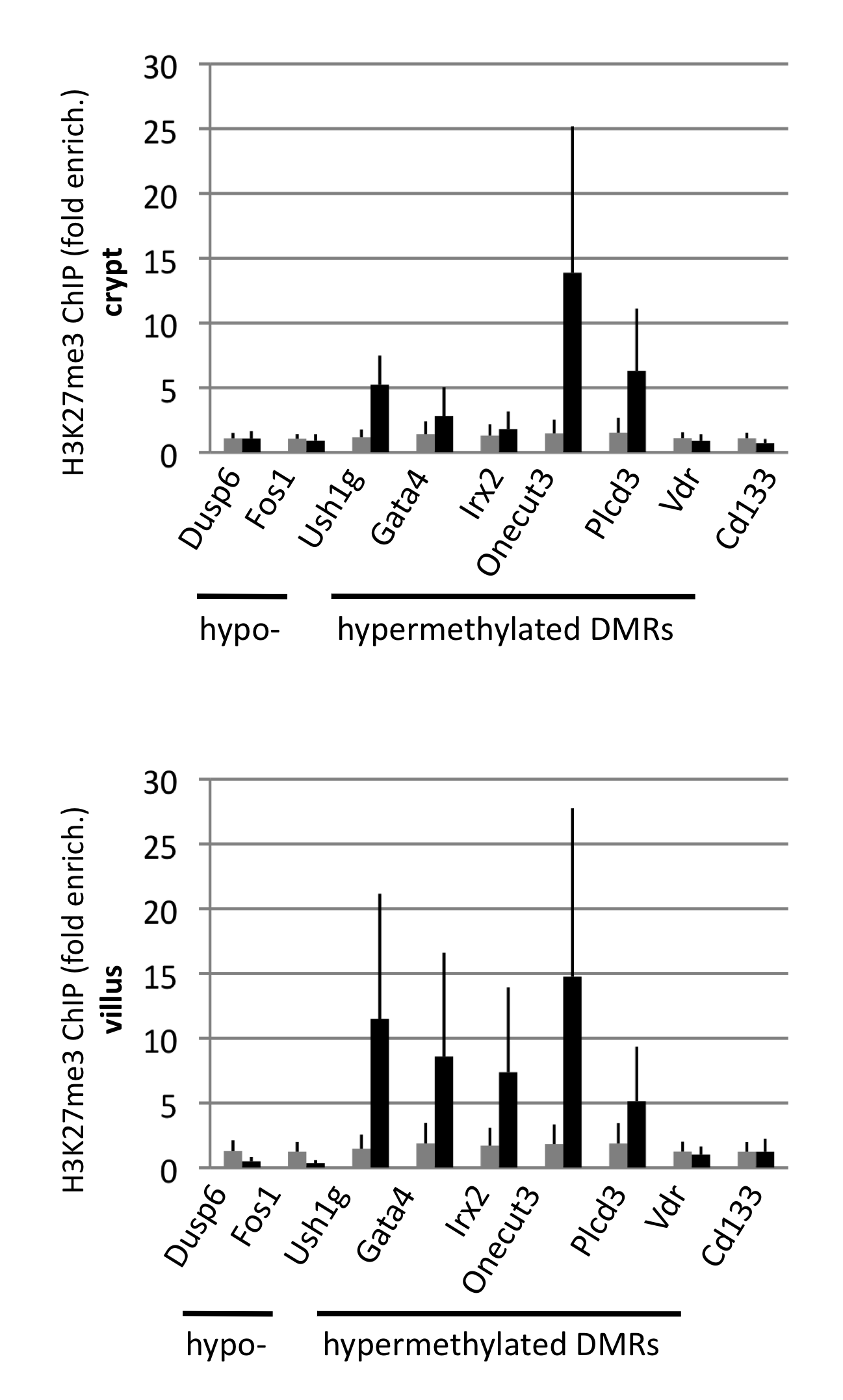

Supplement: Figure S4 — Histone H3K27me3 methylation of DMRs in purified crypt or villus cells. Chromatin was isolated from mouse intestinal crypts (above) or villi (below), and analysed using H3K27me3 chromatin immunoprecipitation, followed by qPCR. black bars: Immunoprecipitated chromatin, grey: Input chromatin. Error bars give standard deviation in three biological replicates. (TIF) [file pgen.1003250.s004.tif]

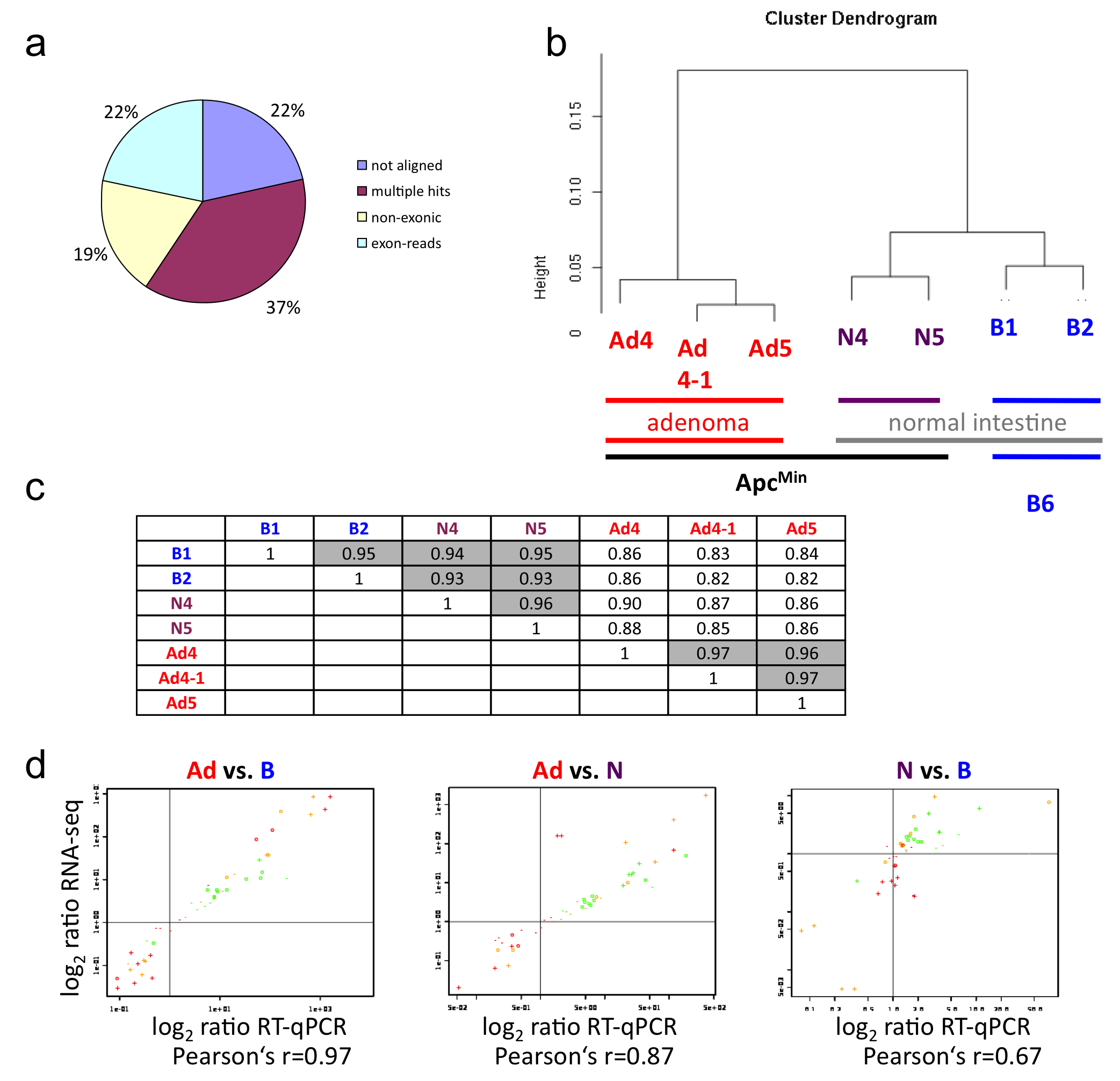

Supplement: Figure S5 — Quality assessment of the RNA-seq data a) RNA-seq read distribution, as percentage of all generated reads. b) Dendrogram showing the hierarchical clustering of the RNA-seq data. c) Spearman's correlation of the RNA-seq samples, as underlying the dendrogram shown in b). The correlations were calculated using those ENSEMBL genes with at least 20 exon read counts in at least one of the samples. rho>0.9 is displayed in grey. The normal intestinal samples of both, ApcMin (N) and B6 (B) are very similar, while most changes in expression are found in adenoma (Ad) d) Validation of the RNA-seq data, by qRT-PCR. Shown are the log2 ratios of the RNA-seq data on the y-axis and the log2 ratios of the qPCR on the x-axis. For the qPCR the same samples that were used for RNA-seq and in addition, four independent samples per group were used. RNA-seq and qRT-PCR are in good agreement. The oligos used for the RT-qPCR experiments are given in Table S10. (TIF) [file pgen.1003250.s005.tif]

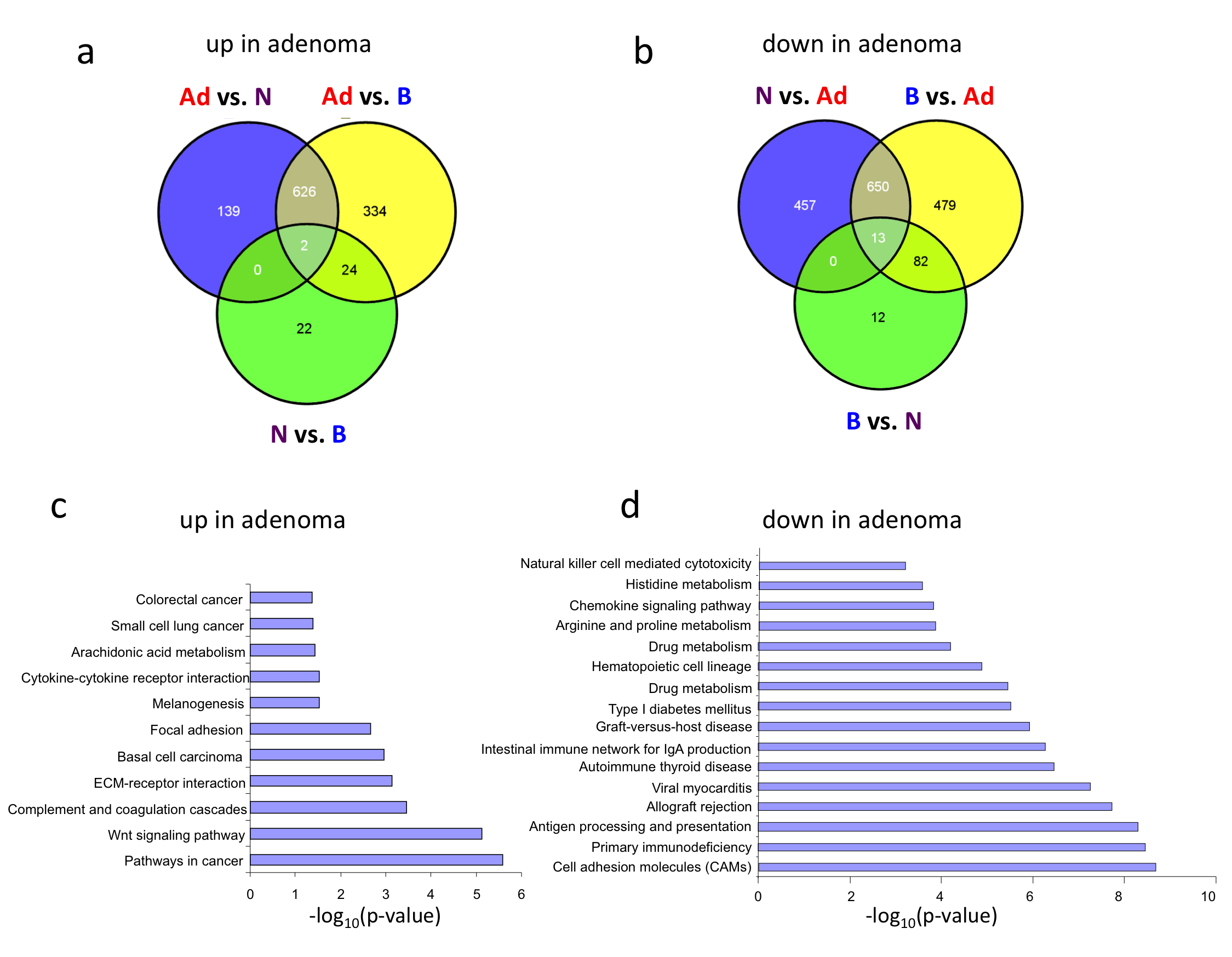

Supplement: Figure S6 — Comparative and KEGG pathway analysis of RNA-seq data. a,b) Venn diagrams display the intersection of differentially expressed genes as determined by edgeR (FDR<0.001) for genes a) up-regulated in adenoma compared to normal intestinal tissue of B6 or APCMin (Ad vs. B, Ad vs. N) and normal intestinal tissue of APCMin compared to normal intestinal tissue of B6 (N vs. B); b) down-regulated in adenoma compared to normal intestinal tissue of B6 or APCMin (N vs. Ad, B vs. Ad) and down-regulated in APCMin normal intestinal tissue compared to B6 (B vs. N). Analyses demonstrate similarity between the normal (B, N) samples, while adenoma (Ad) differs. c,d) KEGG pathway analyses. Overrepresented KEGG pathways in genes up-regulated (c) and down-regulated (d) in adenoma compared to normal intestinal tissue of both, APCMin and B6 as caculated by edgeR (FDR<0.001) are shown. The x-axis displays the −log10 of the p-value calculated by DAVID (http://david.abcc.ncifcrf.gov). Cancer-related pathways are up-regulated in adenoma, while pathways related to immune function are down-regulated. B, normal intestinal tissue from B6; Ad, adenoma from APCMin; N, normal intestinal tissue from APCMin. (TIF) [file pgen.1003250.s006.tif]

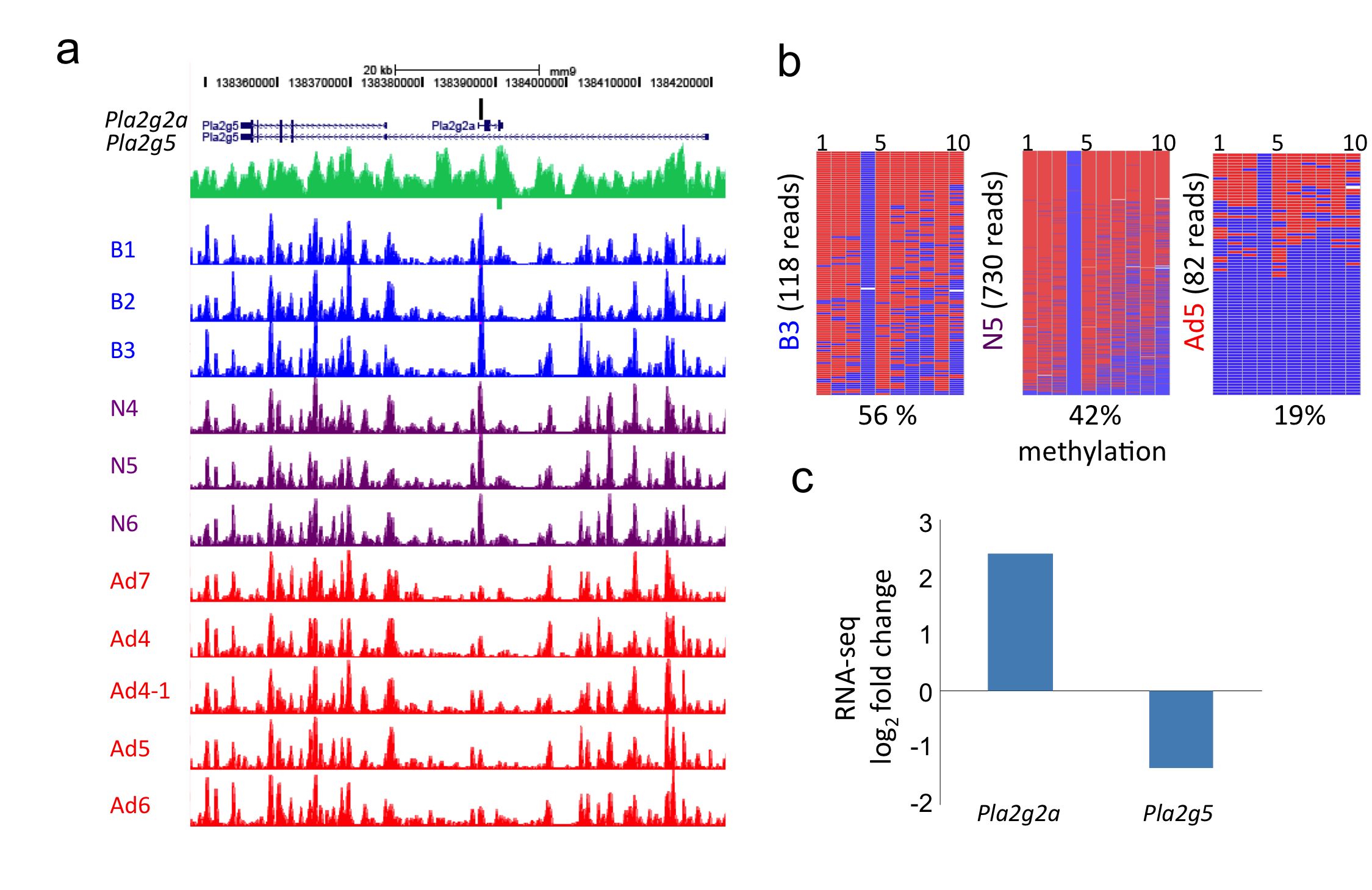

Supplement: Figure S7 — Association of hypomethylation and transcriptional up-regulation in the Pla2g2a locus. a) UCSC browser track of the MeDIP-seq data. The black track displays the identified adenoma-hypomethylated DMR and the position of the validated region. Colour code is as in Figure 1b. b) Heat map of the 454 GS-FLX-based bisulfite pyrosequencing for the Pla2g2a region shown in a. Samples B3, N5 and Ad5 are given. Each column represents a CpG, and each row a generated sequencing read. Red: methylated CpG, blue: unmethylated CpG, white: no data. c) Expression for Pla2g2a and the neighbouring Pla2g5 gene, as determined by RNA-seq. Expression is given as the log2 fold change, as calculated by edgeR for the comparison adenoma vs normal intestinal samples. Both genes are significantly deregulated (FDR<0.001). The elevated expression of Pla2g2a was validated by qRT-PCR using additional samples (data not shown). Pla2g2a is also known as Mom1 (Modifier of intestinal neoplasia 1). (TIF) [file pgen.1003250.s007.tif]

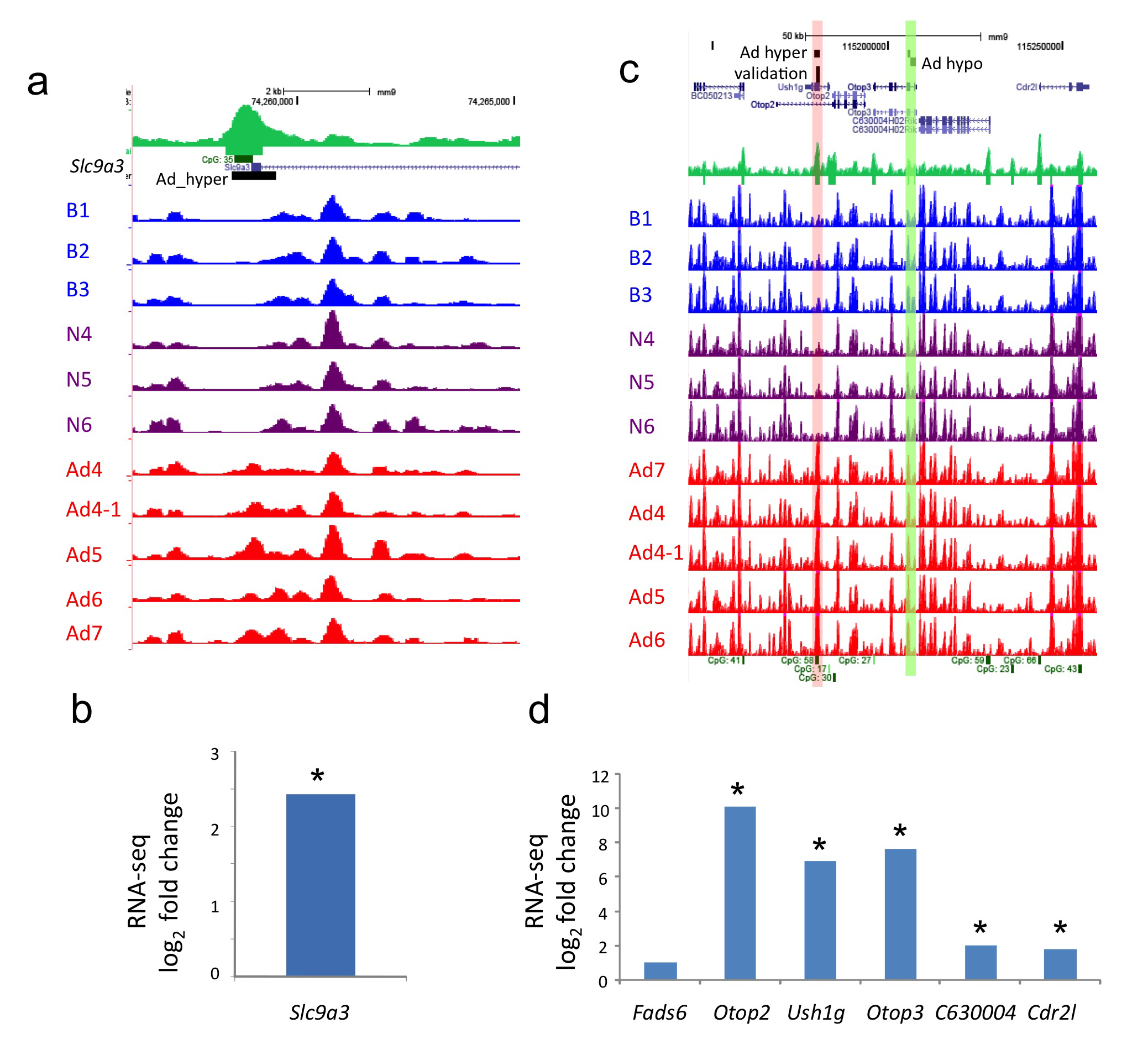

Supplement: Figure S8 — Association of hypermethylation in the Slc9a3 and the Ush1g regions with transcriptional up-regulation. a, b) Promoter hypermethylation in Slc9a3 is associated with transcriptional activation. a) UCSC browser track of MeDIP-seq data. Colour code is as in Figure 1b. b) Expression for Slc9a3 as determined by RNA-seq. Expression is given as the log2 fold change, as calculated by edgeR for the comparison adenoma versus normal intestinal samples. c,d) Methylation marks in the Ush1g region are associated with transcriptional up-regulation of five neighbouring genes within a 90 kb region. c) UCSC browser track of the MeDIP-seq data. The track “Ad hyper” depicts hypermethylated and “Ad hypo” hypomethylated DMRs. Light red overlay: position of a adenoma-hypermethylated DMR, as identified by MeDIP-seq and validated by BS-pyrosequencing; light green overlay: position of a hypermethylated and a hypomethylated DMR next to each other. d) Expression of six adjacent genes (Fads6, Otop2, Ush1g, Otop3, C630004H02Rik and Cdr2l), as determined by RNA-seq. Expression is given as the log2 fold change, as calculated by edgeR for the comparison adenoma versus normal intestinal samples. The differential expression was validated by qPCR for Otop2, Ush1g, Otop3 and C630004H02Rik using additional samples (data not shown). * depicts FDR<0.0000001. (TIF) [file pgen.1003250.s008.tif]
