# Supplementary material for: DNA–Methylome Analysis of Mouse Intestinal Adenoma Identifies a Tumour-Specific Signature That Is Partly Conserved in Human Colon Cancer
Source: PLoS Genet. 2013 Feb 7;9(2):e1003250. doi: 10.1371/journal.pgen.1003250 (PMC3567140; doi:10.1371/journal.pgen.1003250)
Supplement: Text S1 — Supplementary Methods. (DOC) [file pgen.1003250.s019.doc]

**DNA-methylome analysis of mouse intestinal adenoma identifies a tumour-specific signature that is partly conserved in human colon cancer**

Christina Grimm^1,2^, Lukas Chavez^1^, Mireia Vilardell^1^, Alexandra L. Farrall^3^, Sascha Tierling^4^, Julia W. Böhm^4^, Phillip Grote^3^, Matthias Lienhard^1^, Jörn Dietrich^1^, Bernd Timmermann^5^, Jörn Walter^4^, Michal R. Schweiger^1^, Hans Lehrach^1^, Ralf Herwig^1^, Bernhard G. Herrmann^3,6^, Markus Morkel^3,7*^

1 Max Planck Institute for Molecular Genetics, Department of Vertebrate Genomics, Berlin, Germany

2 Charité Universitätsmedizin Berlin, Department of Rheumatology, Berlin, Germany

3 Max Planck Institute for Molecular Genetics, Department of Developmental Genetics, Berlin, Germany

4 Universität des Saarlandes, FR 8.3 Biowissenschaften, Genetik/Epigenetik Campus, Saarbrücken, Germany

5 Max Planck Institute for Molecular Genetics, Next Generation Sequencing Core Facility, Berlin, Germany

6 Charité Universitätsmedizin Berlin, Institute for Medical Genetics, Berlin, Germany

7 Charité Universitätsmedizin Berlin, Laboratory of Molecular Tumor Pathology, Berlin, Germany

* Email: markus.morkel@charite.de

**Supplementary Methods**

**Nucleic Acids Methods**

RNA and DNA Isolation

Mouse normal and adenoma tissue samples were harvested in buffer RLT/1 mM DTT (Qiagen, Hilden, Germany) and homogenized in a Tissue Lyser (Qiagen) for 2 x 2 min, frequency set to 20 with a steel bead of 5 mm diameter (Qiagen). Samples were snap frozen and stored at -80 °C until use.

Library preparation and methylated DNA immunoprecipitation (MeDIP)

To prepare for MeDIP, 8-10 µg genomic DNA were subjected to a RNAse A (Sigma, Taufkirchen, Germany) digest for one hour at 37 °C. Next, DNA was sheared as described previously [1] to a size range of 100-400 bp and purified using Qiaquick PCR columns and buffer QG (Qiagen, Hilden, Germany). Single end libraries were made using 5 µg of fragmented DNA and the genomic DNA sample prep kit (#FC-102-1002, Illumina, San Diego, USA), according to the manufacturer’s instructions with modifications as described previously [2]. In detail, end repair was done in 317 µl total volume with 0.25 mM of each dNTP, 0.1 U/µl T4 DNA polymerase, 0.03 U/µl DNA polymerase I (large fragment) and 0.3 U/µl T4 polynukleotide kinase. A-tailing was performed in the presence of 0.2 mM dATP and 0.5 U/µl Klenow fragment (3’->5’exo-) in a total volume of 88 µl. Adapters were ligated using 29 µl of ‘Adapter oligo mix’ and 10 µl of Quick T4 DNA Ligase in a total volume of 98 µl. All enzymes were purchased from NEB (Frankfurt/Main, Germany). Subsequently, the libraries were used for methylated DNA immunoprecipitation (see below). After MeDIP, the libraries were amplified prior to size selection in a total volume of 30 µl using Phusion High-Fidelity PCR Master Mix with HF buffer (NEB). 20 % of the immunoprecipitated DNA or 40 ng of non-immunoprecipitated library (input) for 6 PCR-cycles. Amplified libraries were run on a 2 % agarose gel (low range ultra agarose, BioRad, München, Germany) and fragments of 150-400 bp were excised (corresponding to insert sizes of 80-330 bp) and purified using the Quiaquick Gel Extraktion Kit (Qiagen). Size-selected libraries were quantified using the QuantIt dsHS Assay Kit on a Qubit fluorometer (Invitrogen, Darmstadt, Germany).

MeDIP was adapted from a previously published protocol [3]. In brief, 10 µl of monoclonal antibody against 5-methylcytidine (#BI-MECY, Eurogentec, Cologne, Germany) were incubated over night with 40 µl Dynabeads M-280 sheep anti-mouse IgG (Invitrogen, Darmstadt, Germany) in 500 µl 0.5% BSA/PBS, washed two times with 0.5% BSA/PBS and one time with IP-buffer (10 mM sodium phosphate (pH7.0), 140 mM NaCl, 0.25 % Triton X100). Prior to immunoprecipitation, the sequencing libraries were denatured for 1 min at 95 °C. 4 µg denatured library was immunoprecipitated for 4 h at 4 °C using the 5-methylcytidine antibody coupled to Dynabeads in a total volume of 230 µl IP-buffer. After immunoprecipitation, the beads were washed three times with 700 µl IP-buffer and then treated with 50 mM Tris-HCl, pH 8.0; 10 mM EDTA, 1 % SDS for 15 min at 65 °C. The supernatant containing the methylated DNA (200 µl) was diluted with 200 µl 10 mM Tris pH 8,0, 1 mM EDTA, treated with proteinase K (0.2 µg/µl) for 2 h at 55°C, followed by phenol-chloroform-extraction and ethanol precipitation. Precipitated DNA was resuspended in 20 µl 10 mM Tris pH 8.5.

Validation of the MeDIP-enrichment by quantitative PCR

Enrichment of methylated DNA was controlled by quantitative PCR. PCR reactions were carried out in 10 µl volume in 384 well plates on a 7900 Fast Real-Time PCR system using SYBR Green PCR master mix (Applied Biosystems, Darmstadt, Germany) using the temperature profile 2 min 50°C, 10 min 95°C, 40 cycles of 15 sec 95°C, 1 min 60 °C followed by a dissociation curve. Relative enrichment was calculated by the ratios of the signals in the immunoprecipitated DNA for a methylated positive and an unmethylated negative control region. For primer sequences, see Table S9.

Preparation of RNA-seq libraries

4 µg of total RNA were depleted for ribosomal RNA using the RiboMinus Euaryote Kit for RNA-seq (Invitrogen) following the manufacturer’s instructions. The RiboMinus depleted RNA was then used for the generation of single end RNA-seq libraries using a strand-specific protocol as described previously [1]. The generated cDNA libraries were size selected on a 2 % agarose gel, fragments of 150-200 nt were excised (corresponding to insert sizes of 80-130 nt) and purified using the Qiaquick Gel extraction kit (Qiagen).

Next generation sequencing

After library quantification with a Qubit device (Invitrogen), a 10 nM stock solution of the amplified library was created. Then, 12 pmol of the stock solution were loaded onto the channels of a 1.4 mm flow cell and cluster amplification was performed. Sequencing-by-synthesis was performed on an Illumina Genome Analyser (GAIIx). All MeDIP and input samples were subjected to 36 nt single read sequencing. The raw data processing was done with the Illumina 1.5 and 1.6 pipelines.

Validation of MeDIP-seq data by bisulfite based methods

*Bisulfite treatment and PCR*

Bisulfite treatment was performed using standard protocols. Briefly, 500 ng genomic DNA was treated with 2 M sodium bisulfite and 0.6 M NaOH. Two thermo spikes of 99 °C for 5 min were introduced followed by two incubation steps of 1.5 h at 50 °C. Purification was achieved by loading, desulfonation and washing on a microcon YM-50 column (Millipore, Schwalbach, Germany). Bisulfite DNA was eluted in 50 µl 1xTE. PCRs for validation of MeDIP-seq data were performed in 30 µl reaction volume in presence of 1 x reaction buffer (10 mM Tris-HCL (pH 8.6), 50 mM KCl, 1.5 mM MgCl_2_), 0.06 mM of each dNTP, 200 nM each, forward and reverse primer, 1.25 U HotStart-IT DNA polymerase (USB, Staufen, Germany) and 2 µl template. Primer sequences and PCR programs are listed in Table S9. Finally, 5 µl of the PCR reaction products were differentiated on a 1.5 % agarose gel.

*SIRPH analyses*

The methylation indices at particular CpGs in MeDIP enriched regions were determined using single-nucleotide primer extension (SNuPE) assays in combination with ion pair reverse phase high performance liquid chromatography (IP RP HPLC) separation techniques (SIRPH). In brief, 5 µl of each PCR product was purified using an ExonucleaseI/SAP mix (1U each, USB, Cleveland, USA) for 30 min at 37 °C followed by a 15 min inactivation step at 80 °C. Then, 14 µl primer extension mastermix (50 mM Tris-HCL, pH9.5, 2.5 mM MgCl_2_, 0.05 mM ddCTP, 0.05 mM ddTTP, 3.6 µM of each SNuPE primer) was added and SNuPE reactions were performed. Obtained unpurified products were loaded on a DNASepTM (Transgenomic, Omaha, USA) column and separated in a primer-specific acetonitril gradient on the WAVETM system (Transgenomic). Methylation indices (MI) were obtained by measuring the peak heights (h) and calculating the ratio h(C)/[h(C)+h(T)]. To confirm the methylation assignment across the DMRs a second CpG position in most amplicons was analyzed in addition. For the SIRPH analyses 25 amplicons were selected and the analyses were performed for seven DNAs that were also used for MeDIP-seq: two adenoma and two normal intestinal samples from B6-Apc^Min^ (Ad and N) and three normal intestinal samples from wild type B6 mice (B).

*Bisulfite pyrosequencing*

454 GS-FLX: Amplicons were generated using region-specific primers with the recommended adaptors at their 5´-end. PCRs were performed in 30 µl reaction volumes in the presence of 10 mM Tris-HCL (pH 8.6), 50 mM KCl, 1.5 mM MgCl_2_, 0.06 mM of each dNTP, 200 nM each, forward and reverse primer, 1.25 U HotStart-IT DNA polymerase (USB, Staufen, Germany) and 2 µl template. For the amplicons BMP1 and ‘T’ the usage of 1.5 U HotStarTaq and Q-Solution (Qiagen, Hilden, Germany) was necessary instead of HotStart-IT to obtain specific PCR products. Specific primer sequences and PCR protocols are provided in Table S9. Amplicons were purified, measured using the Qubit Fluorometer (Invitrogen) and pooled. After emulsion PCR (emPCR), DNA containing beads were recovered, enriched and loaded onto a XLR70 Titanium PicoTiterPlate according to the manufacturer´s protocols. Methylation level and pattern was assessed using multiple sequence alignment with an extended and improved version of the BiQ analyser [4].

First strand cDNA synthesis and quantitative PCR

1 µg of total RNA was reverse transcribed using 200 U of Superscript III and 40 U of RNAseOut in the presence of 1x first strand buffer, 5 mM DTT, 2.5 ng/µl random N6 hexamers, 2.5 µM Oligo dT(20) (Invitrogen) and 0.5 mM of each dNTP (GE Healthcare Europe GmbH, Freiburg ) in a total volume of 20 µl. RNA was denatured for 5 min at 65 °C in the presence of the oligonucleotides and the dNTPs in a total volume of 6 µl. Subsequently, denatured samples were immediately cooled on ice and the reaction mix including the enzymes was added. Samples were incubated for 20 min at 25 °C, 60 min 50 °C followed by a heat inactivation of the enzyme for 15 min at 72 °C. Eventually, 30 µl of 10 mM Tris pH 8.5 was added to the cDNA. Quantitative PCR was carried out in a total volume of 10 µl using 0.33 µl of the diluted cDNA in the presence of 250 nM of each oligonucleotide and 1x SYBR Green PCR master mix in 384 well plates on a 7900 Fast Real-Time PCR system using the default temperature profile followed by the dissociation stage (Applied Biosystems, Darmstadt, Germany). Relative expression was calculated using the ΔΔCt method and *Actb* expression for normalization. Primer sequences are provided in Table S10.

Chromatin immunoprecipitation (ChIP)

Chromatin-immunoprecipitation was carried out according to the standard upstate protocol. Briefly, intestinal tissue was incubated in lysis buffer and mechanical disruption carried out in a 2 ml dounce homogenizer. Lysates were sheared with a Branson W-450 3 mm tip to a 400 bp average fragment size. 10 ug chromatin was incubated with 2 ug of anti-H3K27me3 (Upstate/Millipore) antibody, pre-bound to Protein A magnetic beads (Life Technologies, Darmstadt, Germany). After incubation for 4 h at 4 °C the samples were washed. Reverse crosslinking with Proteinase K was performed overnight at 65 °C. DNA was purified with the Qiagen Enzymatic Reaction Cleanup Kit. Quantitative real-time PCR was carried out on a StepOnePlus qPCR System (Life technologies). An unrelated, intergenic control amplicon (P1: GGAACTTTGCACAGGAGGAG, P2: TGGGTAACGGCTTCCTAATG) was used as normalization control and the relative enrichment over 5 % Input control was determined within the StepOnePlus Software. The data represent six biological replicates. Primer sequences are provided in Table S10.

**Bioinformatics analyses**

Alignment and pre-processing of sequencing reads

Single end sequencing reads (36 bp) generated from MeDIP-seq experiments and input samples were aligned to the mouse genome (UCSC mm9) using Bowtie (version 0.12.5 parameter set -q -n 2 -k 1 --best --maxbts 10000 -m 1) allowing up to 2 nucleotide mismatches to the reference genome per seed and returning only uniquely mapped reads. Replicate sequencing reads (i.e. reads with exactly the same starting position) were counted only once.

The analysis of the MeDIP-seq data was performed with the MEDIPS package [5]. For each sample, the aligned reads were extended in the sequencing direction to a length of 300 nt. The short read coverage of the extended reads was calculated at genome wide 50 bp bins. Subsequently, the final short read count at each genomic bin is transformed into reads per million format (rpm = number of reads in the bin / number of uniquely aligned reads x 1000000) [6].

Identification of differentially methylated regions (cDMRs) between adenoma and normal intestine

Mean rpm values were calculated for genome-wide 500 bp windows overlapping by 250 bp using MEDIPS. Subsequently, for each 500 bp window, we applied a Wilcoxon’s test in order to assess significance of methylation differences between the 5 Apc^Min/+^ adenomas (Ad) versus the 3 Apc^Min/+^ normal intestine (N), 3 Apc^+/+^ wildtype normal intestine (B). Differentially methylated regions (cDMRs) were identified by filtering for 500 bp windows associated with Wilcoxon p-values < 0.01, a ratio of >1.33 or <0.75 and a minimum average signal of 0.25 rpm for one of the two groups. Overlapping significant 500 bp windows were merged if their methylation status indicated an extending DMR.

Annotation of the cDMRs

Each DMR was annotated using ENSEMBL v58 [7]. Annotation included gene structures, transcripts, promoter regions (defined as -1 kb upstream and +500 bp downstream of the transcription start site), exons and introns. Furthermore, CpG islands were identified using the CpG island searcher on the repeat masked mm9 genome [8] and the following criteria: GC content above 50 %, CpG observed to expected ratio >0.6, length >300 bp. CpG island shores were defined as 1 kb regions upstream or downstream of a CpG island, respectively. DMRs were annotated with repetitive regions using the repeat masker table provided by UCSC. DMRs overlapping conserved elements were identified using the table browser function of the UCSC genome browser (mm9) and the Vertebrate Elements track [9-11].

RNA-seq analysis

36mer RNA-seq reads were aligned to the mouse genome using Bowtie (version 0.12.5 – parameter set: -n 2 -l 36 -y --chunkmbs 256 --best --strata -k 1 -m 1) against the genomic reference UCSC mm9. Subsequently, reads that did not map to the genome were aligned to the cDNA reference ENSEMBL v58 in order to map reads spanning exon junctions. Then, uniquely mapped reads aligning to the sense strand of a gene were counted. Differential expression was calculated using the R/BioConductor edgeR package[16](#_ENREF_16), cut-off FDR≤0.001.

Gene Set enrichment Analysis

Gene set enrichment analyses (GSEA) were performed following the author’s guidelines [12]. Genes were ordered according to rpm-normalized MeDIP-seq or RNA-seq read counts. For promoter methylation, reads encompassing -1.0 to +0.5kb were counted. For gene body methylation, reads were counted according to gene annotation in ENSEMBL v58. Gene signatures used are given, along with references, in Table S6. False discovery values were calculated from 1000 gene set permutations. Significance cut-offs were FDR<0.1, P<0.05.

Comparison of DNA methylation and RNA expression

MeDIP-seq sequencing reads were counted in the promoter regions (-1 kb to 500 bp) or in the gene bodies of ENSEMBL v58 genes. Differentially methylation in promoter regions and in the gene bodies was assessed with the R/BioConductor edgeR package[16](#_ENREF_16), cut-off p<0.01. Differential methylated promoter or gene body regions were compared to genes identified as differentially expressed as described under RNA-seq analysis.

Comparison of human and mouse data

The mouse orthologues to the human ENSEMBL58 IDs were identified using BioMart. Human ENSEMBL IDs were used to identify the corresponding mouse ENSMBL IDs, resulting in a list of 15055 unique orthologues (i.e. for one human ID only one mouse ID exist and vice versa). For 14336 genes RNA-seq reads were obtained for both organism.

Comparison of MeDIP-seq derived methylation to bisulfite-based methods

Genomic regions validated by SIRPH and bisulfite pyrosequencing were defined as regions of interest (ROIs). The MEDIPS software package [5] was applied to the MeDIP-seq data in order to estimate reads per million (rpm) normalized to library size and the CpG density dependent relative methylation scores (rms) for each tested ROI.

**Supplementary Methods References**

1. Parkhomchuk D, Borodina T, Amstislavskiy V, Banaru M, Hallen L, et al. (2009) Transcriptome analysis by strand-specific sequencing of complementary DNA. Nucleic Acids Res 37: e123. doi:10.1093/nar/gkp596.

2. Grimm C, Adjaye J (2012) Analysis of the methylome of human embryonic stem cells employing methylated DNA immunoprecipitation coupled to next-generation sequencing. Methods Mol Biol 873: 281–295. doi:10.1007/978-1-61779-794-1_19.

3. Weber M, Davies JJ, Wittig D, Oakeley EJ, Haase M, et al. (2005) Chromosome-wide and promoter-specific analyses identify sites of differential DNA methylation in normal and transformed human cells. Nat Genet 37: 853–862. doi:10.1038/ng1598.

4. Bock C, Reither S, Mikeska T, Paulsen M, Walter J, et al. (2005) BiQ Analyzer: visualization and quality control for DNA methylation data from bisulfite sequencing. Bioinformatics 21: 4067–4068. doi:10.1093/bioinformatics/bti652.

5. Chavez L, Jozefczuk J, Grimm C, Dietrich J, Timmermann B, et al. (2010) Computational analysis of genome-wide DNA methylation during the differentiation of human embryonic stem cells along the endodermal lineage. Genome Res 20: 1441–1450. doi:10.1101/gr.110114.110.

6. Mortazavi A, Williams BA, McCue K, Schaeffer L, Wold B (2008) Mapping and quantifying mammalian transcriptomes by RNA-Seq. Nat Methods 5: 621–628. doi:10.1038/nmeth.1226.

7. Flicek P, Amode MR, Barrell D, Beal K, Brent S, et al. (2012) Ensembl 2012. Nucleic Acids Res 40: D84–D90. doi:10.1093/nar/gkr991.

8. Takai D, Jones PA (2003) The CpG island searcher: a new WWW resource. In Silico Biol 3: 235–240.

9. Kent WJ, Sugnet CW, Furey TS, Roskin KM, Pringle TH, et al. (2002) The human genome browser at UCSC. Genome Res 12: 996–1006. doi:10.1101/gr.229102.

10. Karolchik D, Hinrichs AS, Furey TS, Roskin KM, Sugnet CW, et al. (2004) The UCSC Table Browser data retrieval tool. Nucleic Acids Res 32: D493–D496. doi:10.1093/nar/gkh103.

11. Fujita PA, Rhead B, Zweig AS, Hinrichs AS, Karolchik D, et al. (2011) The UCSC Genome Browser database: update 2011. Nucleic Acids Res 39: D876–D882. doi:10.1093/nar/gkq963.

12. Subramanian A, Tamayo P, Mootha VK, Mukherjee S, Ebert BL, et al. (2005) Gene set enrichment analysis: a knowledge-based approach for interpreting genome-wide expression profiles. Proc Natl Acad Sci USA 102: 15545–15550. doi:10.1073/pnas.0506580102.
